# Advanced Soft Robotic System for In Situ 3D Bioprinting and Endoscopic Surgery

**DOI:** 10.1002/advs.202205656

**Published:** 2023-02-19

**Authors:** Mai Thanh Thai, Phuoc Thien Phan, Hien Anh Tran, Chi Cong Nguyen, Trung Thien Hoang, James Davies, Jelena Rnjak‐Kovacina, Hoang‐Phuong Phan, Nigel Hamilton Lovell, Thanh Nho Do

**Affiliations:** ^1^ Graduate School of Biomedical Engineering Faculty of Engineering UNSW Sydney Kensington Campus Sydney NSW 2052 Australia; ^2^ Tyree Institute of Health Engineering UNSW Sydney Sydney NSW 2052 Australia; ^3^ School of Mechanical and Manufacturing Engineering Faculty of Engineering UNSW Sydney Kensington Campus Sydney NSW 2052 Australia

**Keywords:** endoscopic surgery, feedforward control, in situ bioprinting, soft robotics

## Abstract

Three‐dimensional (3D) bioprinting technology offers great potential in the treatment of tissue and organ damage. Conventional approaches generally rely on a large form factor desktop bioprinter to create in vitro 3D living constructs before introducing them into the patient's body, which poses several drawbacks such as surface mismatches, structure damage, and high contamination along with tissue injury due to transport and large open‐field surgery. In situ bioprinting inside a living body is a potentially transformational solution as the body serves as an excellent bioreactor. This work introduces a multifunctional and flexible in situ 3D bioprinter (F3DB), which features a high degree of freedom soft printing head integrated into a flexible robotic arm to deliver multilayered biomaterials to internal organs/tissues. The device has a master‐slave architecture and is operated by a kinematic inversion model and learning‐based controllers. The 3D printing capabilities with different patterns, surfaces, and on a colon phantom are also tested with different composite hydrogels and biomaterials. The F3DB capability to perform endoscopic surgery is further demonstrated with fresh porcine tissue. The new system is expected to bridge a gap in the field of in situ bioprinting and support the future development of advanced endoscopic surgical robots.

## Graphical abstract

This work introduces a multifunctional and flexible in situ 3D bioprinter that features a high degree of freedom soft printing head integrated into a flexible snake‐like robotic arm to deliver multilayered biomaterials or perform endoscopic surgery. The new system will bridge a gap in the field of in situ bioprinting and support the future development of advanced endoscopic surgical robots.

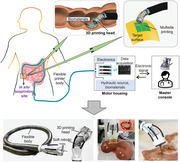

## 1 Introduction

Every year, millions of people around the world suffer from tissue damage due to disease, trauma, injury, and as a consequence of surgery.^[^ ^1^ ^]^ For surgical procedures, sutures are mostly used to promote tissue healing. However, failure of wound closure or repair of defects in the gastrointestinal (GI) tract, blood vessels, or other organ surfaces can lead to unexpected complications, including infections.^[^ ^2^ ^]^ Gastric wall injury is one of the most common diseases in GI tract, which is due to the weakness of the mucosae created by H. pylori or bleeding. Typical treatment for this disease mainly includes medication and surgery. While the efficacy of the medication method is normally slow, surgery is associated with complications. Although the use of styptic colloid through spraying with an endoscope is currently used to stop bleeding, this method is not able to reconstruct the 3D structure of the wound. For patients with cardiovascular diseases, the death of myocardium (e.g., cardiomyocytes) can compromise cardiac muscle contraction, leading to cardiac dysfunction and finally chronic heart failure.^[^ ^3^ ^]^ Recently, 3D bioprinting technology with biomaterials incorporating living cells (bioinks) and drugs has emerged as an excellent method to create 3D living constructs (e.g., cardiac patch or GI patch) for the treatment of a variety of conditions, such as myocardial infarction.^[^ ^4^ ^]^ 3D bioprinting approaches also have the potential for many other biomedical applications including sutureless repair of the GI defect, the restoration or facilitation of the healing of damaged tissues and organs, biomolecule delivery, and regenerative medicine.^[^ ^5^ ^]^ Currently, 3D live constructs are created outside the human body where they are either incubated in vitro for maturation before implantation or externally 3D printed and then implanted in vivo using large open‐field surgery.^[^ ^5^, ^6^ ^]^ Over the last decades, desktop 3D bioprinters with large‐form factors are at the core of all commercially available and research‐grade bioprinting approaches.^[^ ^7^ ^]^ Notable desktop bioprinters include NovoGen MMX (Organovo, Delaware, USA), 3D Discovery (RegenHU, Swiss), INKREDIBLE (Cellink, Swiss), BIOBOT TM, and BIOASSEMBLYBOT (Advanced Solutions, USA), BIO3D (Singapore), or RASTRUM 3D (Inventia Life Science, Australia).^[^ ^8^ ^]^ One of the major challenges with these desktop 3D bioprinters is the mismatch between externally printed live constructs and a target tissue surface during the implantation process. As biomaterials are normally made from soft and fragile structures, structural damage can occur during the manual handling, transferring, and transport process.^[^ ^5^, ^6^ ^]^ High contamination risks due to the direct exposure to the fabrication platform and the surrounding environment together with the requirement of a strictly sterile environment during the printing process are also major issues. In addition, external 3D printing environments are not comparable with the living body which serves as an excellent bioreactor for biomaterials. A large open‐field surgery required for the introduction of printed materials into the body can lead to a longer recovery time and a higher medical cost.^[^ ^6a^ ^]^

To overcome these challenges associated with the in vitro incubation process, poor bioreactor, and surface mismatch, in situ bioprinting techniques where living biomaterials are directly deposited onto target tissues have risen as a promising solution.^[^ ^5^ ^]^ One of the simplest approaches is the use of a handheld tool that directly deliver biomaterials into defect tissue.^[^ ^9^ ^]^ Although this method allows in situ bioprinting, it is limited to damaged areas at or near external skin surfaces or accessible sites that require large open‐field surgery. The handheld tools also limit access to many other internal organs and tissues such as heart, colon, intestine, and kidney and in turn pose a high infection risk and longer recovery time.^[^ ^10^ ^]^ In addition, the hand‐held tools are manually controlled, which results in low precision of printed structures or low speed of material generation. Recently, Urciuolo et al.^[^ ^11^ ^]^ introduced a new bioprinting approach where living biomaterial was directly injected into areas at or near the skin's surface. Near‐infrared light was then delivered through the skin to induce photoresponsive crosslinking of the biomaterials. Although this injection method offers minimally invasive benefits, it has shallow penetrability of the light, which is insufficient for crosslinking biomaterials, and therefore it is only limited to printing sites where depth is less than 5 mm, which is not suitable for many internal tissues and organs such as colon, heart, or blood vessels. Zhao et al.^[^ ^12^ ^]^ introduced a proof‐of‐concept of a bioprinting system for gastric injury treatment. This system used onboard rigid DC motors and stiff linkages to create the printing head, which made the device bulky and unsuitable for minimally invasive delivery. In addition, it was not equipped with a flexible bending arm and therefore it had poor flexibility and a small printing area due to the occlusion caused by the rigid components. Zhou et al.^[^ ^13^ ^]^ recently developed a soft robotic needle capable of in situ computer‐controlled printing. The authors used four large permanent magnets driven by complex DC motor systems to magnetically create 2D deflection of the needle tip where the translation in the axial direction was achieved by an external DC motor. Despite advances, this approach required large permanent magnets and complex magnetic shielding to control the needle tip, with only access to the target site via a skin incision. In addition, the bending control for the tip was limited due to the use of external magnetic fields, which are ill‐suited to operate near ferromagnetic materials or reach complex paths and sites within the human body or via the human natural orifice.^[^ ^14^ ^]^

**Figure 1:**
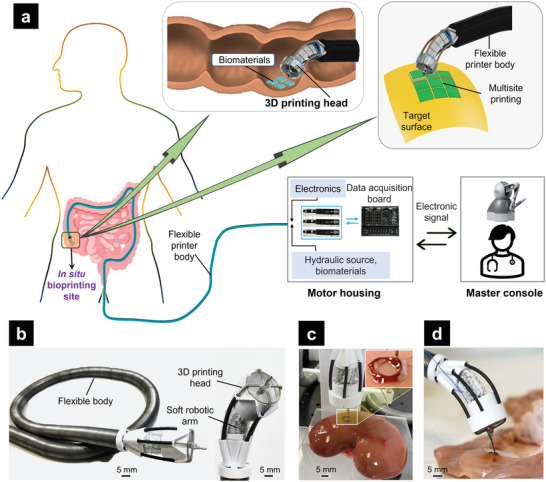
Flexible 3D bioprinter (F3DB) system. a) Schematic of the F3DB that can perform in situ bioprinting on the surface of internal organs and tissues such as the intestine, stomach, or heart at multiple locations. The whole system is driven by soft artificial muscles via an external hydraulic source (motor housing) where the user steers the printing tip via the master console. b) A prototype of the F3DB with its flexible body, 3D printing head, and flexible robotic arm. c) Ex vivo 3D bioprinting of biomaterial on a fresh porcine kidney. d) Tissue dissection on fresh porcine colon using the printing nozzle as an electrosurgery knife.

To address the above shortcomings, this paper reports a novel, miniaturized, and flexible 3D bioprinter (F3DB) that can directly deliver multilayered biomaterials onto the surfaces of internal organs and tissues. Our disruptive technology features a high degree of freedom (DOF) printing head and a soft robotic arm which are mounted onto a long and flexible snake‐like body. The device can potentially access confined and hard‐to‐reach areas within the living bodies via small skin incisions or the human natural orifices (e.g., mouth, anus). The new F3DB is expected to overcome several major barriers from existing 3D bioprinting technologies by i) removing the need for in vitro incubation of living materials for maturation before surgical implantation, ii) avoiding the interface mismatches between the printed biomaterials and target surfaces, and iii) providing a small printing device footprint. The F3DB shares a similar architecture with existing flexible surgical systems via a master‐slave configuration (**Figure** **1**). The soft robotic arm with three DOFs of bending and extension motions is developed using advanced functionalities in soft hydraulic actuators, while the 3D printing head which features three DOFs of motion is mounted on the soft robotic arm to form a complete 3D bioprinter. The device is fully actuated using soft artificial muscles and controlled by a kinematic inversion model, a new nonlinear hysteresis model, and a machine learning‐based controller. The workspace, frequency response, durability, and force generation of the F3DB are experimentally validated. The printing capabilities are evaluated with a fresh porcine kidney and artificial colon using various materials such as food‐grade chocolate, liquid silicone elastomer, gel composite, and biomaterial (X‐Pure GelDAT with a high density of L929 living cells) with different patterns and surfaces.

## 2 Results

### 2.1 Overall Working Principle

The F3DB system is designed with a master‐slave architecture where the user at the master console remotely manipulates a slave manipulator consisting of a soft robotic arm and a 3D printing head. The slave manipulator is directly mounted onto a long and flexible catheter that serves as a flexible printer body (Figure 1a,b). Once the 3D printing head has reached the target site, an automatic control algorithm based on an inverse kinematic model (Note S2, Supporting Information) is enabled to induce the motion of the soft robotic arm and the printing nozzle in three directions where multilayered biomaterials are delivered onto the surfaces of internal organs or tissues at multiple locations. The whole system is driven by hydraulic soft microtubule artificial muscles (SMAMs)^[^ ^15^ ^]^ and soft fabric bellow actuators (FBAs). The master console mainly includes a haptic interface (Geomatic Touch Haptic Systems, 3D Systems, USA) which transmits the user's hand motion to the motor housing (Note S1, Supporting Information) for remotely steering the soft robotic arm. To monitor the printing site with real‐time videos, we used a flexible miniature camera with an outer diameter of 1.66 mm (MISUMI Electronics Corp, Taiwan).

**Figure 2:**
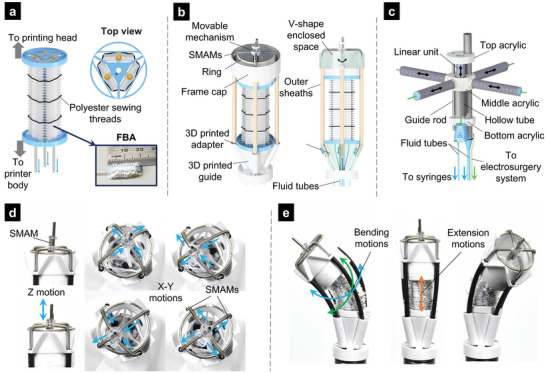
Detailed design of the slave manipulator of the F3DB. a) 3‐DOF soft robotic arm. b) Schematic illustration of the soft robotic arm and 3D‐printing head with an integrated nozzle at the tip. c) The movable mechanism consists of a linear unit in combination with the four SMAMs that can provide 3D motion for the nozzle. d) Motions of the nozzle integrated into the 3D printing head in three directions. e) Bending and extension motions of the robotic arm.

The motion range of the slave manipulator is shown in Table S2 (Supporting Information). While the active soft robotic arm provides omnidirectional bending for coarse navigation toward the region of interest (Figure 1), the nozzle of the 3D printing head offers 3‐DOF fine movements in front of the targeted surface (**Figure** **2**). The motor housing (Figure S5, Supporting Information) remotely actuates the soft artificial muscles of the slave manipulator using linear hydraulic units and micro‐sized fluid transmission tubes. Leveraging the advantage of hydraulic pressure, the F3DB can be comprised of any length to reach long and dexterous paths within the body such as the GI tracts, which is a contrast to cable‐driven mechanisms which have high nonlinearity, friction, and force loss.^[^ ^16^ ^]^ The 3D printing head can automatically operate under predefined trajectories, while the biomaterials are delivered by a syringe dispenser. Once the printing head completes the first printing task at one location, the soft robotic arm will be steered to reach other locations and the printing process is resumed, enabling multisite printing (Figure 1a). With this feature, the printing area can be expanded to cover whole surfaces of internal organs or tissues (e.g., colon, stomach, heart, and bladder) which is unachievable with existing in vivo bioprinting devices (Table S1, Supporting Information). It is worth noting that the F3DB including the slave manipulator is scalable and therefore it can be designed and fabricated to suit specific bioprinting needs. In this work, we fabricated a robotic arm with an outer diameter of 20 mm, length of 50 mm, and weight of ≈100 g, which can be potentially inserted into a GI tract via a natural orifice or delivered minimally invasively via a small skin incision. To demonstrate its scalability, we also fabricated a smaller version of the F3DB with an outer diameter of 11.5 mm and a working area of 9 mm as shown in Figure S11 (Supporting Information). This prototype has a similar diameter to commercial therapeutic endoscopes (outer diameter in the range of 11.3–13.2 mm^[^ ^17^ ^]^), which is small enough to be potentially inserted into a GI tract via a natural orifice or a small skin incision. For the smaller prototype, we utilized three SMAMs as a soft robotic arm of 7 mm outer diameter instead of FBAs to optimize the overall size, so the F3DB prototype can be further scaled down if needed. A typical operating process of the F3DB to reach the internal printing target is shown in Movie S1, Supporting Information.

### 2.2 Design and Fabrication

#### 2.2.1 Soft Robotic Arm

The 3‐DOF soft robotic arm is actuated via three FBAs which are arranged in parallel and at the vertices of an equilateral triangle to enable omnidirectional motion of the printing head (Figure 2). The bending movement of the arm is regulated by the hydraulic pressures inside the FBAs which are transmitted from external linear hydraulic units via micro‐sized PTFE tubes (Cole‐Parmer, USA). To map the motion of plungers from the external hydraulic syringe to the elongation of each FBA for 3‐DOF tip movement, a kinematic model was developed and employed (Notes S1 and S2, Supporting Information). For example, actuating a single FBA will induce bending movement in one direction while operating two FBAs creates bending movement in a plane in between the two bellow axes. Similarly, when all three FBAs simultaneously receive the same hydraulic pressure, a linear translational motion is achieved along its axial axis. This actuation mechanism allows the soft robotic arm to automatically extend its length without moving the flexible printer body, which is critical for most cable‐driven surgical robots where external translation motion which is highly associated with nonlinear hysteresis and friction is required.^[^ ^18^ ^]^

Each FBA consists of an inner elastic silicone tube and an outer constraint bellow made from wrinkled, nonstretchable fabric layers. Once the fluid is supplied into the inner elastic tube, there is an increase in its internal volume and the circumferential constraints imposed by the fabric layers cause a lengthening along the axis of each tube, with no radial expansion. In this state, elastic energy is stored in the structure. As the fluid pressure or volume is decreased, the stored elastic energy is released to yield a contraction force which is useful for tissue cutting or ablation when the F3DB is used as a surgical tool. While increased hydraulic pressure produces a lengthening of the FBA, generated forces are normally produced via contraction, analogous to human biological muscles.^[^ ^19^ ^]^ The fabrication process of each FBA is presented in Note S3 and Figure S2a (Supporting Information). To form the soft robotic arm, three FBAs are triangularly arranged in parallel and connected to 3D‐printed adaptors. While the top adaptor connects to the 3D printing head, the bottom adaptor links to the flexible printer body. By changing the FBA design and parameters, it is possible to control the manipulator's range of motion and its overall outer diameter. A prototype of the FBA shown in Figure 2a; and Figure S2b (Supporting Information) is 20 mm in length, and 10 mm in width, and can achieve up to 100% elongation under hydraulic pressure. The FBA can be fabricated at any length and scaled, while its structure stiffness can be tuned by using different types of elastic tubes, fabrics, and wrinkling levels.

#### 2.2.2 3D‐Printing Head

The 3‐axis printing head is directly mounted onto the tip of the soft robotic arm (Figure 2b,c). This mechanism has a similar working principle as that of conventional desktop 3D printers, which facilitates the motion of a nozzle in three directions. The printing head consists of four soft microtubule artificial muscles (SMAMs), each has an outer diameter of Ø1.2 mm, a housing frame (Ø20 mm), a movable mechanism consisting of a hollow nozzle, acrylic connectors, and a guide rod that provides translational motion of the nozzle perpendicular to the 3D printing head, and outer sheaths. Details on the design and fabrication of SMAM can be found in our previous work.^[^ ^20^ ^]^ One of the advantages of using these soft artificial muscles to drive the nozzle is that they can maintain constant energy efficiency (fixed nonlinear hysteresis profile) when working against long and dexterous paths, eliminating complex compensation control compared to cable‐driven mechanisms where the nonlinear hysteresis profiles always change with the varying working paths.

**Figure 3:**
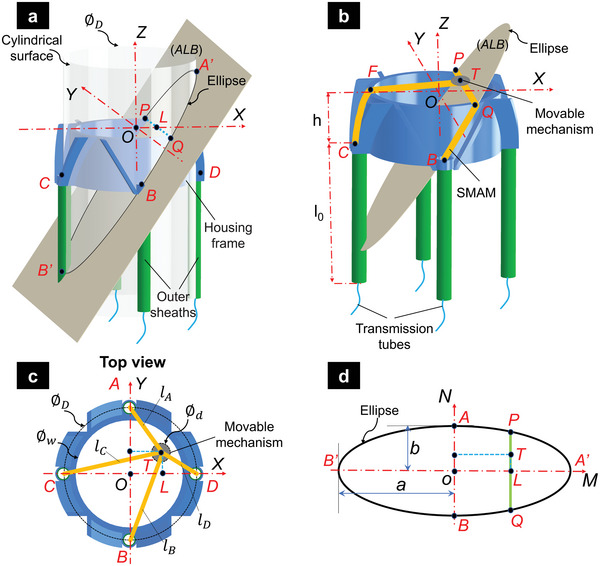
Geometric illustration of the 3D printing head where a central cylinder intersects with the plane (*ALB*). a,b) Schematic diagram of the 3D printing head with an ellipse at the intersection. c) The position of the movable mechanism on the *XOY* plane. d) Top view of the ellipse, which is an intersection of plane *ALB* and the cylindrical surface passing six points (*A*, *A’*, *B*, *B’*, *C*, and *D*).

To control the nozzle, four SMAMs are arranged in a cross shape and connected to the movable mechanism via a 4‐way connector (middle acrylic, Figure 2c). The detailed fabrication process of the 3D printing head is shown in Note S4 (Supporting Information). By controlling the length of each SMAM, the motions of the printing nozzle in the *XOY* plane (**Figure** **3**) can be achieved via an inverse kinematic model which describes the relationship between the instantaneous length of SMAMs and the position of the printing nozzle. The motion in the Z‐axis of the printing nozzle is independently controlled by another SMAM. With this design, the nozzle can flexibly move within the housing frame of an inner diameter of ∅_*w*_= 17 mm (Figure 3c). To avoid the sharp bend occurring at contacting points between SMAMs and the housing frame,^[^ ^20b^ ^]^ each SMAM freely moves inside a V‐shape enclosed space (Figure 2b). A low‐friction ring located at the top of a 3D‐printed frame (Figure 2b) is also used to guide the soft muscle when working against a bending curve of 90^°^. To maximize the working range of the nozzle, we selected a nominal length ( *l*_0_ = 30 mm) for each SMAM, which is larger than the diameter of the housing frame. To avoid buckling and motion effects, each SMAM is guided to slide inside an outer sheath (Figure 2b). The fluid transmission tubes for SMAMs are routed along the robotic arm to minimize the system's overall size.

To provide the Z‐axis motion for the printing nozzle, a miniature linear unit within the movable mechanism was developed (Figure 2c). The linear unit was designed to maximize the printing area while enabling a strong force to puncture through tissue (e.g., lifting agent injection or performing cutting and coagulating with an electrosurgery unit in case it is used for endoscopic submucosal dissection (ESD)). The movable mechanism consists of a printing nozzle (diameter of 1.25 mm), a guide rod (diameter of 0.48 mm), and a hollow tube (diameter of 1.77 mm) which enables the internal movement of a SMAM (diameter of 1.27 mm and length of 8 mm). Briefly, once this SMAM is hydraulically pressurized, it pushes the top acrylic element upward (Figure 2c,d). A guide rod was used to constrain the nozzle motion perpendicular to the *XOY* plane. When the hydraulic pressure drops, the contraction force of the SMAM brings the top acrylic element backward. The linear unit could achieve a 6 mm extension and exert 1.5 N of axial force. It is also worth noting that the nozzle and linear unit are movable and therefore their fluid transmission tubes should be as flexible as possible so that they do not affect their motion. In this work, we fabricated a helical polytetrafluoroethylene (PTFE) hydraulic tube with an outer diameter of 0.6 mm, which was then connected to the SMAM. To transmit low‐pressure agents such as bioinks, water, or lifting agent to the printing nozzle, a soft silicone tube (outer diameter of 1.2 mm) was used. The fabrication method for these elements is shown in Figure S3 and Note S4 (Supporting Information).

### 2.3 Inverse Kinematic Model of the Nozzle

In this work, four SMAMs are used to control the nozzle position in the *XOY* plane, while another SMAM is employed to independently actuate the nozzle motion in the Z‐axis direction (Figure 2). To control the position of the nozzle, an inverse kinematic model that represents the relationship between the movable mechanism *T*(*X*, *Y*) and the entire length of each SMAM (*l_A_*,*l_B_*,*l_C_*,*l_D_*) was developed. It is noted that each SMAM was initially pressurized to reach a certain length that is longer than its nominal one (at zero hydraulic pressure). The schematic diagrams for the 3D printing head are depicted in Figure 3b,c where *A*, *B*, *C*, and *D* are the central points of SMAM outlets and *O* is the central point of the movable mechanism with a diameter ∅_*d*_ (when the four SMAMs are at the resting state). The plane *ALB* passes through three points (*L*(*X*, 0) in *XOY*‐coordinate, *A* and *B*) and intersects with a cylinder of diameter ∅_*D*_ containing four points *A*, *B*, *C*, and *D* to form an ellipse (Figure 3d) with a central point *o*(*0,0*). The points *P*, *T*, and *Q* are collinear, lying on a single line which is the intersection between the plane *XOY* and the plane *ALB*. The point *o*(*0,0*) is the central point of the line through *A* and *B*. The semimajor axis with a length of *a* and the semiminor axis with a length of *b* of the ellipse can be determined by

(1) $$\begin{array}{c} \left\{\begin{array}{c} a=\sqrt{\frac{P_{M}^{2}}{1-\frac{P_{N}^{2}}{b^{2}}}}\\ b=\frac{\emptyset_{D}}{2} \end{array}\right.\mathrm{with}\left\{\begin{array}{c} P_{M}=oL=\sqrt{OL^{2}+h^{2}}=\sqrt{X^{2}+h^{2}}\\ P_{N}=PL=\sqrt{OP^{2}-OL^{2}}=\sqrt{{\left(\frac{\emptyset_{D}}{2}\right)}^{2}-X^{2}}\\ X<\frac{\emptyset_{D}}{2},Y<\frac{\emptyset_{D}}{2} \end{array}\right. \end{array}$$

where *P_M_*,*P_N_* are the projection of *P* on the *M*‐axis and *N*‐axis of the plane *MoN*, respectively; *h* is the distance between the plane (*ABC*) and the plane (*XOY*).

The entire length of two opposite SMAMs (*l_A_*,*l_B_*) passing through two points *A* and *B* (Figure 3b,c) can be calculated by

(2) $$\left\{\begin{array}{c} l_{A}=l_{0}+AP+PT-0.5\emptyset_{d}=l_{0}+AP+P_{N}-Y-0.5\emptyset_{d}\\ l_{B}=l_{0}+BQ+QT-0.5\emptyset_{d}=l_{0}+BQ+P_{N}+Y-0.5\emptyset_{d} \end{array}\right.$$

(3) $$\begin{array}{ccc} AP=BQ & = & \int_{0}^{P_{M}}\sqrt{1+{\left(\frac{dN}{dM}\right)}^{2}}dM\\ & = & \int_{0}^{P_{M}}\sqrt{1-\frac{4M^{2}X^{4}}{\emptyset_{D}^{2}\left(\frac{4M^{2}X^{2}}{\emptyset_{D}^{2}\left(h^{2}+X^{2}\right)}-1\right){\left(h^{2}+X^{2}\right)}^{2}}}dM \end{array}$$

where *l*_0_ is the length of the SMAM inside the outer sheath.

Similarly, the entire length (*l_C_*,*l_B_*) of the other SMAMs is expressed by

(4) $$\left\{\begin{array}{c} l_{C}=l_{0}+CF+F_{M}+X-0.5\emptyset_{d}\\ l_{D}=l_{0}+CF+F_{M}-X-0.5\emptyset_{d} \end{array}\right.$$

(5) $$\left\{\begin{array}{c} F_{M}=\sqrt{{\left(0.5\emptyset_{D}\right)}^{2}-Y^{2}}\\ F_{N}=\sqrt{Y^{2}+h^{2}}\\ CF=\int_{F_{M}}^{0.5\emptyset_{D}}\sqrt{1+{\left(\frac{dN}{dM}\right)}^{2}}dM=\int_{F_{M}}^{0.5\emptyset_{D}}\sqrt{1-\frac{4M^{2}\left(Y^{2}+h^{2}\right)}{\emptyset_{D}^{2}Y^{2}\left(\frac{4M^{2}}{\emptyset_{D}^{2}}-1\right)}}dM \end{array}\right.$$

The value of the geometric parameters is shown in Table S3 (Supporting Information).

### 2.4 Characterization and Position Control

**Figure 4:**
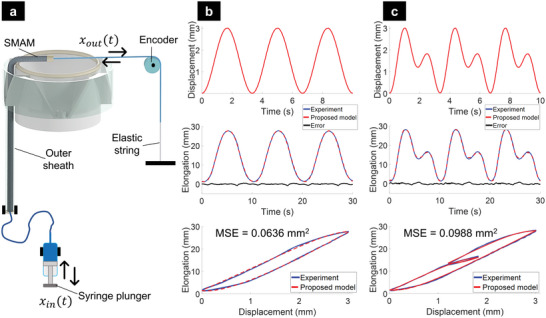
Hysteresis profile of a single SMAM within the 3‐axis printing head and results of the proposed hysteresis model with different input signals. a) Experimental setup. b) 0.3 Hz sine wave excitation. c) A dual‐frequency sinusoidal excitation (combined frequencies of 0.3 and 0.6 Hz).

Before implementing a control algorithm into the 3D printing head, we first characterized the motion of a single SMAM within the printing head to understand the open‐loop repeatability, which is an important metric for reproducing identical motions for the nozzle. We applied cyclic input signals (the displacement of the syringe plunger) and recorded the output motion (i.e., elongation of SMAM). The experimental setup (**Figure** **4**a) was created with a similar structure as that of the 3D printing head, but only a single SMAM was tested. It is worth noting that this experiment could fully represent the real characteristics of the soft muscle when they are integrated into the 3D printing head. In this work, we used different input signals to reveal the hysteresis profile of the SMAM. Experimental results (Figure 4b,c) show that the SMAM hysteresis was asymmetric for both loading and unloading phases (e.g., increased or decreased hydraulic pressures). The hysteresis loop was wider in the middle and significantly narrower toward the reverse points. The presence of hysteresis due to memory effects, nonlinear friction, and elastic deformation of soft materials means that the SMAM length in the 3D printing head highly depends on the time history of the input motion and therefore achieving a precise position for the printing nozzle with an open‐loop control is challenging.

To deal with such nonlinearity, this paper introduces two main control approaches, which offer better tracking performance of the printing nozzle. The first method is a model‐based approach, which employs a feedforward compensation in combination with the kinematic model. To utilize this method, a hysteresis model for soft artificial muscle was first derived and identified from a set of input/output (I/O) measurements and then a feedforward controller was computed based on the identified parameters. The second method is a data‐driven algorithm or a machine learning‐based controller where no precise mathematical model is required for the nonlinear compensation. Although the model‐based control approach requires less computational resources and time to derive the model parameters compared to the machine learning‐based controller, it is well suited for a system where no external disturbances or model uncertainties are presented. In contrast, the machine learning‐based controller can deal with the nonlinearities and uncertainties originating from system variations and external environments. However, it requires a huge amount of data to train the controller. Therefore, depending on the specific application which requires a certain level of accuracy, the hysteresis model‐based controller (e.g., endoscopic surgery), or machine learning‐based controller (e.g., precise 3D bioprinting) can be flexibly selected.

#### 2.4.1 Hysteresis Modeling and Feedforward Compensation

Due to the effect of nonlinear hysteresis, the SMAM elongation is not a simple linear function of the input position from the syringe plunger, but instead, it depends on the input syringe position, its history, and motion direction. To minimize the hysteretic effects, a feedforward‐based controller that requires a nonlinear hysteresis model was developed. A typical approach to capture the hysteresis profile of a system is the use of integral models such as Preisach, Maxwell‐Slip, and Prandtl–Ishlinskii (PI)^[^ ^21^ ^]^ where discrete state‐space models are mostly used. Alternatively, differential models such as the Bouc–Wen model and its variants have been shown to be excellent candidates for capturing the dynamic hysteresis behaviors via continuous state space and shape variables of the hysteresis loops.^[^ ^22^ ^]^ Despite low complexity for both implementation and computation, there is a trade‐off between the number of model variables and accuracy, which are normally associated with the offline identification process. In this work, we introduced a new asymmetric hysteresis model that can effectively capture the nonlinear hysteresis profile of the SMAMs in the 3‐axis printing head. Compared to the symmetric Bouc–Wen model and the generalized asymmetric hysteresis Bouc–Wen model,^[^ ^15^ ^]^ our new asymmetric hysteresis model offers a fewer number of model parameters, higher accuracy, and less computational time (Note S5, Supporting Information). We define the SMAM displacement output as Φ_*S*_(*x*, *t*) = *x*_out_ (*t*) and the displacement input as *x*( *t*) = *x*_in_ (*t*) (Figure 4a), a hyperbolic tangent is incorporated into the hysteresis state space model to smoothen the reverse curve at the transition point of the hysteresis loop. The new model is expressed by

(6) $$\Phi_{S}\left(x,t\right)=\left\{\begin{array}{c} 0:x(t)<0\\ \alpha_{x1}\tanh(x\left(t)/\alpha_{x2}\right)+\alpha_{z}z\left(t\right):x\left(t\right)\geq 0 \end{array}\right.$$

(7) $$\dot{z}\left(t\right)=\left|\dot{x}\left(t\right)\right|\;\left[A\mathrm{sgn}(\dot{x}\left(t)\right)-\upsilon {\left|z(t)\right|}^{n-1}z\left(t\right)+\rho \right]$$

The dimensionless parameters *A*, υ, *ρ*, and *n* in Equations (6) and (7) control the shape and size of the hysteresis loops. The coefficients *α*_*x*1_, *α*_*x*2_, and *α*_*z*_ represent the ratio of output elongation Φ_S_(*x*,*t*) to the input displacement *x*(*t*) and the internal state *z*(*t*). By minimizing the mean square error (*MSE*) between the model output and the measured experimental data based on Particle Swarm Optimization (PSO), seven parameters are identified and optimized. To validate our new hysteresis model, different desired trajectories (Figure 4a) were used. The identified model parameters using PSO were *α*_*x*1_= 38.81, *α*_*x*2_= 3.20, *α*_*z*_= ‐0.13, *A* = 34.54, υ = 1.01, *ρ* = 4.43, and *n* = 1.05, and the identification results are shown in Figure 4b,c.

**Figure 5:**
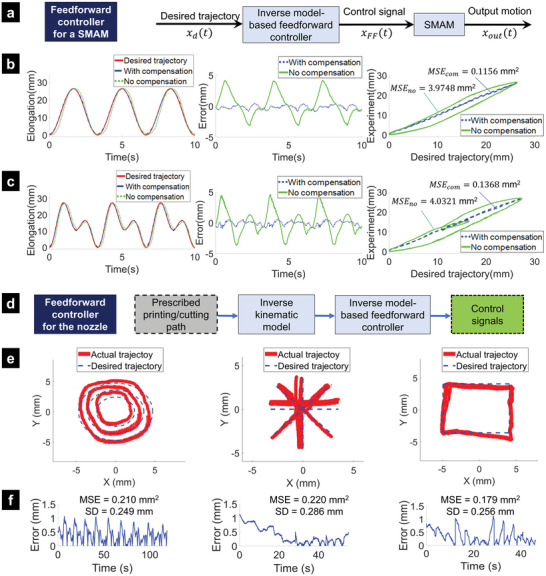
Inverse hysteresis model‐based feedforward controller and validated results. a) Diagram of the feedforward controller for a SMAM. b) Position tracking results for a single SMAM using a single frequency reference (0.3 Hz). c) Position tracking result for a single SMAM using a periodic reference (combination of 0.3 and 0.6 Hz). d) Diagram of the feedforward controller for the printing nozzle where four SMAMs are simultaneously controlled. e) Tracking results for the printing nozzle trajectories with the feedforward controller where four SMAMs are simultaneously actuated. f) Corresponding tracking errors.

The goal of feedforward compensation is to utilize a direct inverse model to approximately eliminate the hysteresis loop. In this work, we used an inverse multiplicative method (**Figure** **5**a) for the new hysteresis model given by Equations (6) and (7). To implement this approach, several assumptions were made: i) initial length and fluid pressure for the SMAMs were kept the same for both the identification process and compensation control; ii) output position feedback which was used for offline identification was not available during the compensation process. We then commanded the different desired trajectories to the SMAM shown in Figure 4a and compared the uncompensated and compensated outputs. The results from Figure 5b,c revealed that there was a higher tracking error between the desired trajectory *x*_d_(*t*) and the measured output *x*_out_(*t*) if the feedforward controller was not implemented. Quantitatively, the tracking performance for the case of a single frequency (0.3 Hz) had an *MSE*_no_= 3.9748 mm^2^ (without compensation) and *MSE*_com_= 0.1156 mm^2^ (with compensation). For the periodic reference (combination of 0.3 and 0.6 Hz), the *MSE*_no_ = 4.0321 mm^2^ without compensation and *MSE*_com_= 0.1368 mm^2^ if the compensation was engaged.

To precisely control the position of the printing nozzle to follow any desired trajectories, a feedforward controller based on the inverse of the new hysteresis model for four SMAMs was proposed. First, predefined paths of the nozzle were planned and the length of each SMAM was derived by using the inverse kinematic model given by Equations (1)–(5). Next, the SMAM lengths were fine‐tuned by the hysteresis model‐based feedforward compensation as the high‐level layer (Figure 5d). It is noted that the linear units comprising DC motors and internal proportional–integral–derivative (PID) controller were exploited to generate controlled motions at syringe plungers as the low‐level layer. To validate our approach, we used different profile trajectories such as concentric circles, straight lines, and rectangular paths (Figure 5e). To capture the nozzle position for comparison purposes, we used computer vision techniques that process the real‐time images captured by a Logitech HD Pro Webcam Black C920 (Figure S5, Supporting Information) in MATLAB (MathWorks Inc., USA). We also plotted the tracking results for the printing nozzle and compared them with desired trajectory as well as the associated tracking error (Figure 5f; and Movie S5, Supporting Information). Results showed that the tracking error from the hysteresis compensation for experiments with individual SMAMs is smaller than that of combined SMAMs in the 3D printing head. It can be explained by the interaction force between SMAMs during the operation but this unlikely affects the effectiveness of the hysteresis model‐based feedforward compensation. It is also worth noting that a smaller tracking error with feedforward compensation requires higher accuracy of the identified model parameters for the hysteresis loop.

#### 2.4.2 Machine Learning‐Based Controller

**Figure 6:**
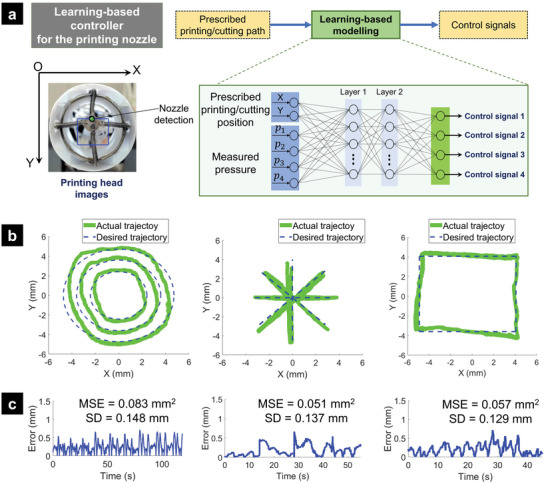
Architecture of proposed imaging system and the machine learning‐based controller. a) Overview of the machine learning‐based control scheme. b) Trajectory tracking with different profiles for the printing nozzle using the machine learning‐based controller. c) Corresponding tracking errors.

To provide a better tracking performance (Figure 5e) under the presence of disturbances (e.g., interaction between SMAMs), we also introduced a new machine learning‐based controller. The new method was combined with the inverse kinematic model given by Equations (1)–(5), which established a mapping from printing nozzle movements on the projection printing plane to the actuation of the printing tip actuation. It is noted that the machine learning‐based control approach (**Figure** **6**a) required a sufficient amount of training data sets to approximate the physical structure. Therefore, the proposed machine learning‐based controller was trained with time‐varying 2D printing nozzle position (X, Y) and SMAMs pressure (*p*_1_, *p*_2_, *p*_3_, *p*_4_) as inputs and the displacement of syringe plungers, *l*_1_, *l*_2_, *l*_3_, *l*_4_) as outputs. To create the datasets, the printing nozzle was commanded to follow various patterns such as concentric circles, rectangles, and spirals with a sampling rate of 100 Hz. The developed inverse kinematic model given by Equations (1)–(5) was used to roughly calculate the input motions of syringe plungers from linear motion units. The printing nozzle position was recorded and processed by a Logitech HD Pro Webcam Black C920. One thousand datasets were collected and randomly split into training/validation/testing sets in the ratio of 75/15/15, respectively. A neural network (NN) with two hidden layers and Bayesian regularization backpropagation was applied to train the network parameters. We used the Dynamic Time‐series function in MATLAB and one delay sample to train the controller where an Intel Xeon E‐2136 @ 3.30 GHz was used. The trained learning‐based model approximately represented both the inverse kinematics of the robot and the nonlinearity of SMAMs. When implementing the trained NN to specific paths (concentric circles, rectangles, and straight lines), the desired printing/cutting path and current SMAMs pressure were continuously inputted into the trained model and the output signals were fed to the DC motors to pump water in and out of the inner channels of the SMAMs. This machine learning‐based control approach is an offline‐learning controller and the precise trajectory tracking highly depends on the training model of the datasets.

The tracking results given in Figure 6b–c; and Movie S5 (Supporting Information) show that the printing nozzle could follow the concentric circles, the straight lines, and the rectangular paths, which commonly comprises other, more complex patterns, with MSE < 0.083 mm^2^ and Standard Deviation (SD) < 0.148 mm. These excellent results also suggest that the printing tip paired with the machine learning‐based model could produce repeatable and accurate paths even with open‐loop control. Compared to the hysteresis model‐based feedforward controller, the machine learning‐based control method provided better tracking results, but it took a longer time for training and processing. However, there still exists a tracking error due to the use of an open‐loop controller. Therefore, a closed‐loop controller with an advanced nonlinear hysteresis model such as the one in^[^ ^23^ ^]^ and online adaptive feedback as shown^[^ ^16a^ ^]^ should be considered in future work.

### 2.5 3D Bioprinting Demonstrations

#### 2.5.1 Ex Vivo Tests of the F3DB with Various Materials

**Figure 7:**
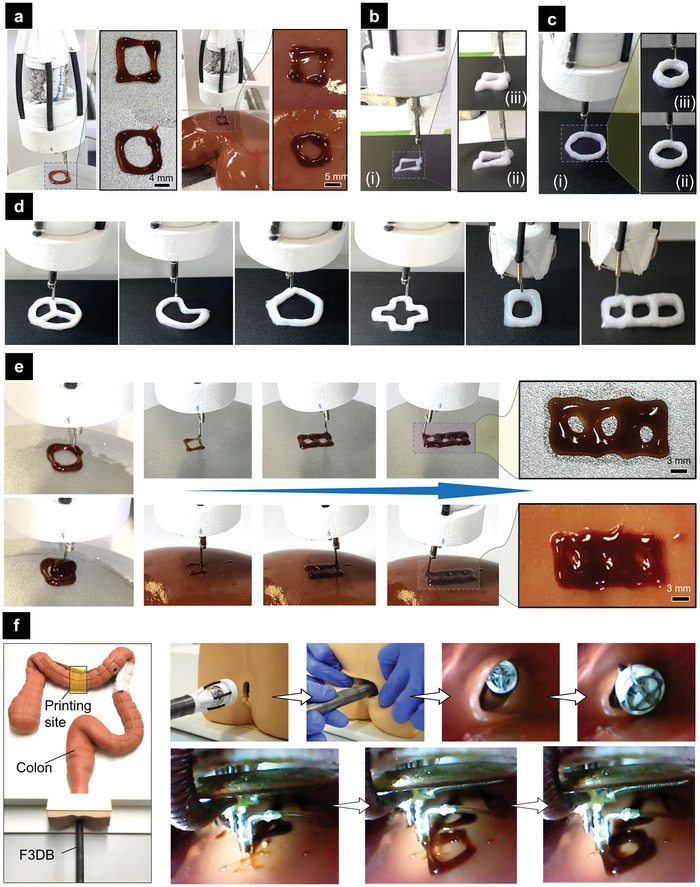
Printing performance of the F3DB with different materials and shapes on various surfaces. a) Printing of liquid chocolate with three‐layered rectangular and circular shapes on a flat surface (left panel) and fresh porcine kidney (right panel). b,c) 3D printing of gel composite made from cationic polymers, silicones, alcohol, and olive oil on a flat surface with three layers i), five layers ii), and seven layers iii). d) 3D printing of gel composite on a flat surface with different shapes and layers. e) Circular printing of liquid chocolate on a flat surface with concentric filling (left panel) and multisite printing of liquid chocolate (right panel) on a flat surface (top figures) and a fresh porcine kidney (bottom figures). f) In situ 3D printing of two‐layered liquid chocolate inside a colon phantom (transverse colon segment). (Top right panel) Insertion process of the F3DB into the colon channel to reach the target site within the transverse colon segment via the anal canal and rectum. (Bottom right panel) In situ printing process of the two‐layered rectangular shape onto the inner surface of the colon phantom.

To demonstrate the capability of the F3DB to work with various biomaterials, experiments were validated with various materials such as liquid food‐grade chocolate and composites of liquid silicone elastomers (e.g., Ecoflex 30 series). It is noted that the 3D‐printed liquids were chosen arbitrarily, due to their ease of printing. Different 3D printed patterns with multilayers such as three‐layered circles, three‐layered rectangles, five‐layered rectangles, or combined seven‐layered rectangles and concentric circles were employed. It is worth noting that the resolution of the printed filament highly depends on five main parameters: i) the speed of moving nozzle, ii) the feeding speed of the material, iii) the inner diameter of the nozzle, iv) the distance from the nozzle to the printing target, and v) the viscosity of the materials. As shown in Figure S13 (Supporting Information), a faster moving velocity usually stretches the printed filament while a slower speed results in a larger size for the filament; while increasing the material flow increases the filament thickness. Furthermore, while the larger inner size of the nozzle significantly expands the extruded fiber, reducing the distance from the nozzle to the printing target slightly makes the printing filament larger. In addition, in 3D printing resolution refers to the fineness of the details that can be printed in the final object. It is typically measured in terms of layer thickness, which is the thickness of each layer of material that is laid down during the printing process. The resolution of a 3D printer is determined by a variety of factors, including i) the size of the nozzle, ii) the accuracy of the printing head, iii) printing speed, and iv) the properties of the material being used. As shown in Figure S15 (Supporting Information), while the larger inner size of the nozzle significantly increases the thickness of the extruded fiber, reducing the printing speed makes the printing filament thicker. In this work, the materials were supplied to an interchangeable printing nozzle with various inner sizes (0.5–0.9 mm) by a miniature syringe dispenser with a capacity of 1 mL and the feeding speed was set as 0.1 mm s^−1^. This feeding speed could be adjusted and optimized after the printing results were evaluated and a smaller nozzle size would allow us to print with a higher resolution. The layer thickness and the distance from the nozzle to the printing target could be controlled by the *Z*‐axis actuator or a SMAM in the movable mechanism with the feedforward controller to obtain mean square error or *MSE*_com_= 0.1156 mm^2^. To provide precise motion for the nozzle in the *X‐Y* axis, the movable mechanism was controlled by a machine learning‐based algorithm (Section 2.4.2) with MSE < 0.083 mm^2^ and Standard Deviation (SD) < 0.148 mm. The properties of the material will be further investigated in future works. **Figure** **7**a shows the printing results of three‐layered rectangular and circular shapes with liquid chocolate on the surfaces of a flat substrate and a fresh porcine kidney respectively. We also conducted 3D printing of three, five, and seven layers of a gel composite made from cationic polymers, silicones, alcohol, and olive oil, respectively (Figure 7b,c). To demonstrate our system capability, we also performed multilayered and multisite 3D printing of the new gel composite on various shapes such as triradiate pattern, pie pattern, pentagon pattern, and cross pattern (see Figure 7d). Additional 3d printing results can be also found in Figure S14, Supporting Information. To demonstrate the capability of our F3DB to conduct infill printing, we also conducted concentric printing where the liquid chocolate was concentrically printed and filled to form a circle (Figure 7e). All printing processes in Figure 7 can be found in Movie S2, Supporting Information). One of special features of our F3DB is scalability, meaning that it can be designed to suit specific bioprinting needs and at desired size. We, therefore, fabricated a smaller version of the F3DB with an outer diameter of around Ø 11.5 mm for the printing head. We then perform 3D printing with the same gel composite in five‐layered circular shapes, five‐layered rectangular shapes, and multisite printing on a flat surface. The printing results are presented in Figure S12, Supporting Information.

Given its advantages of 6‐axis printing and slender nature, the F3DB was also validated with multisite printing where various patterns were 3D printed at multiple locations (Figure 7e; and Movie S2, Supporting Information). One of the advantages of multisite printing is that it allows the F3DB to have a smaller size for minimally invasive delivery (via small skin incision) or noninvasive delivery (via natural orifices) to reach internal tissue or organs while maintaining the printing capability over a larger surface area. It is worth noting that the 3‐DOFs soft robotic arm provides omnidirectional bending for coarse navigation toward the region of interest, the 3D‐printing head creates highly precise patterns and thicknesses of multiple layers via a compensation controller (see previous sections). During the printing process at each printing location, only the distal printing head is actively moved, which ultimately accounts for the precision of the system at that location. To steer the printing head of the F3DB to reach other locations, the robotic arm is manually manipulated via a haptic interface (Geomatic Touch Haptic Systems, 3D Systems, USA) using the developed kinematic model (Note S2, Supporting Information) in order to ensure that a correct alignment between printed profiles at different locations is achieved.

To demonstrate the capability of our F3DB to perform in situ 3D printing directly on internal organs and tissues, we also conducted in situ 3D printing of two‐layered food‐grade chocolate within the transverse segment of a colon phantom (Colonoscopy Trainer Model #2003, The Chamberlain Group, USA). In practice, the use of a flexible camera which is routed along the F3DB body will be used to monitor the printing process. For proof‐of‐concept and validation purposes, this experiment used a miniature camera which was installed in front of the experimental setup to better capture the printing site. The deployment process for the F3DB is shown in the top right panel of Figure 7f where the F3DB is inserted to reach a printing target within the transverse colon segment via the anal canal and rectum while the printing process is given in the bottom right panel of Figure 7f. The real‐time performance of this in situ printing is also shown in Movie S1 (Supporting Information).

#### 2.5.2 Ex Vivo Bioprinting with Living Biomaterial

**Figure 8:**
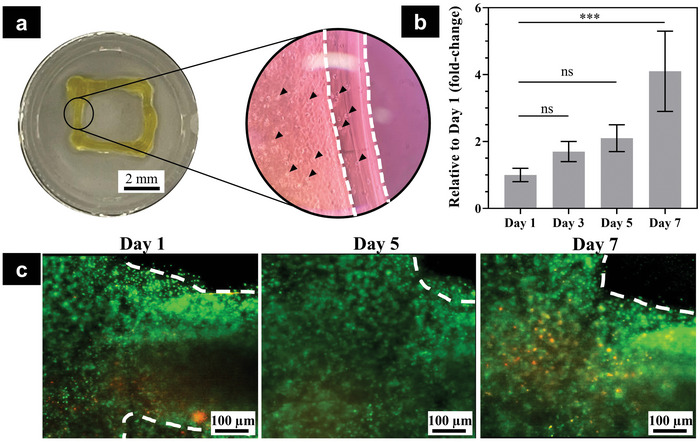
F3DB supported extrusion‐based cell bioprinting as potential in situ bioprinting. a) Representative image of the printed construct at a macro scale (left) and microstructure of printed construct using phase‐contrast microscopy (right). Note that the white dashed lines showed printed layers and black arrows represented cells in the printed structure. b) Cell metabolic activity expressed as fold‐change relative to day 1 postprinting. The data are presented as mean ± standard deviation (*n* = 4). c) Representative images of live/dead staining. Live cells are green and dead cells are red.

To further demonstrate the feasibility of our technology, we also tested the developed F3DB with living biomaterial where an extrusion‐based cell printing setup was used. The main aim was to examine the cell viability after being printed via an F3DB nozzle and a long fluid transmission tube. In this experiment, we used the same F3DB setup as that of previous experiments. The microstructure of printed live constructs was observed using phase‐contrast microscopy and cell viability was studied for seven days postprinting using Alamar blue assay and live/dead fluorescent staining. The biomaterial with living cells or bioink was prepared (see the Experimental Section). Briefly, the bioink that consisted of a commercial ink (X‐Pure GelDAT with a high density of L929 cells (1.4 × 10^6^ cells mL^−1^)), supplemented medium and photoinitiators (i.e., tris (bipyridine) ruthenium (II) chloride and sodium persulfate) was directly printed on a flat substrate. To monitor the cell viability, we created a bilayer of rectangular cell printed constructs (**Figure** **8**a). Experimental results revealed that the cells were not affected by the printing process as the majority of cells were alive postprinting, and the number of cells constantly increased by fourfold on day 7 postprinting as compared to day 1 (Figure 8b,c).

It is noted that the printed constructs were slightly changed over time as compared to the freshly printed live construct due to the cell spreading and proliferation over time. Furthermore, gelatin and its modified products such as gelatin methacryloyl have a high degradation profile which could be degraded quickly within several days to weeks.^[^ ^24^ ^]^ Consequently, the bioink, GelDAT, and tyrosine‐modified gelatin might also perform the same degradation properties as others. In addition, due to the limitation of the screening and scanning process to acquire the same fluorescent image areas, the dashed lines on day 1 were slightly different from day 5 and day 7. However, we would like to highlight that this paper mainly focuses on the design, fabrication, and evaluation of a novel and flexible in situ 3D bioprinter, capable of delivering different gel materials onto desired surfaces to benefit existing emerging biomaterials. Therefore, the promising results confirmed that F3DB could be used as a flexible bioprinter for in situ bioprinting applications without damaging the living cells.

#### 2.5.3 An Application of F3DB for Endoscopic Surgery

**Figure 9:**
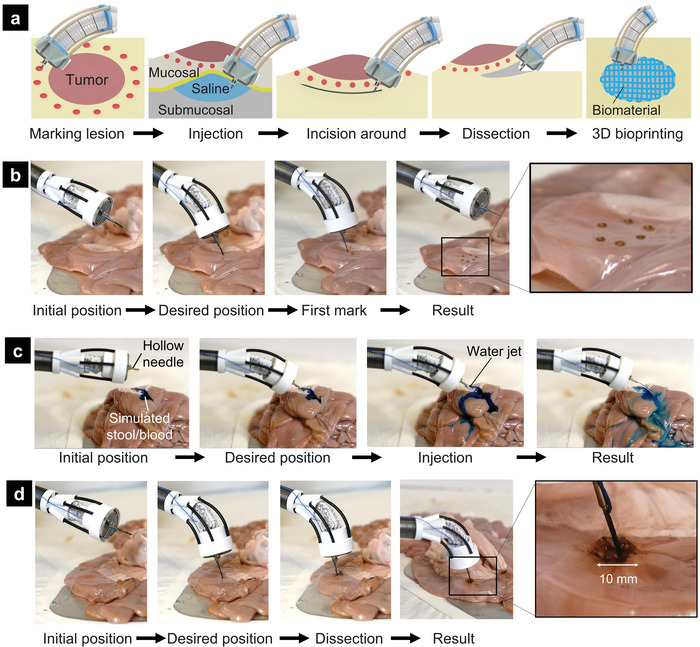
Working principle of F3DB for endoscopic surgery. a) Illustration of endoscopic submucosal dissection (ESD) procedure with F3DB. b) Demonstration of marking lesion perimeter with electrosurgery. c) Demonstration of washing lesion surface. d) Demonstration of the circular dissection in a lesion.

Worldwide, colorectal cancer (CRC) is the third most common cause of cancer death.^[^ ^25^ ^]^ Early removal of colorectal neoplasia leads to an increase of at least 90% in the patient's 5 year survival rate.^[^ ^26^ ^]^ It has been shown that endoscopic submucosal dissection (ESD) achieves a high rate of en bloc resection (84–95%) with a lower rate of local recurrence (≈1%) for the management of laterally spreading tumors and greater margins for more accurate histological examination of the resected specimen.^[^ ^26b^ ^]^ Existing endoscopic robots require multiple changeable tools for water injection, cleaning, lesion marking, and dissection. This increases procedural times and contamination risks.^[^ ^14^ ^]^ In this work, we demonstrated that our F3DB which can be used as an all‐in‐one endoscopic surgical tool was also capable of performing multiple tasks that potentially add benefits to the ESD procedure. A typical workflow for an ESD procedure is illustrated in **Figure** **9**a. First, the F3DB tip is inserted toward a targeted location by an endoscopist through natural orifices such as the mouth or anus. Second, the flexible robotic arm and 3D printing head are steered to mark the perimeter of the lesion via the printing nozzle which is connected to an electrosurgery unit (Figure 9b). Third, a lifting agent (saline) is injected into the submucosa around the perimeter by the nozzle tip. It is noted that the printing nozzle should be sharpened for ESD purposes. Fourthly, the 3D printing head in combination with the motion of the flexible robotic arm cuts circumferentially around the target lesion using the printing nozzle as an electrosurgical knife. To clean stool or blood that occurred during the submucosal dissection, water will be also delivered via the nozzle. To facilitate faster tissue healing after the surgery, 3D printing of living biomaterial can be directly performed (Section 2.5.1 and Figure 7f).

To validate the capability of our F3DB, we experimentally tested it on fresh porcine intestine (a detailed experimental setup is shown in Figure S6a, Supporting Information). Figure 8b–d demonstrates the use of F3DB as an all‐in‐one endoscopic surgical tool that could perform various functions such as marking lesion perimeter (Figure 8b), washing lesion surface with water jet (Figure 8c; and Movie S3, Supporting Information), and circular dissection (Figure 8d; and Movie S3, Supporting Information). During the experiment, the bending motion of the robotic arm was regulated by the master console, the circular dissecting function was executed under a predefined program based on the learning‐based controller. Regarding its working procedure, the arm was initially fixed and then bent toward the desired position before performing desired surgical tasks. Once the dissection is completed, the robotic arm and the printing head will return to their initial position before conducting the ESD procedure at other desired targets. These ex vivo results show that our F3DB is a promising candidate for the future development of an all‐in‐one endoscopic surgical tool for ESD procedures.

## 3 Discussion and Conclusion

3D bioprinting is a new method that enables the controlled assembly of biomaterials with living cells into complex living structures to restore the function of damaged tissues/organs such as heart, blood vessels, trachea, and intestine. Despite advances, existing 3D bioprinting techniques require the fabrication and maturation of tissue constructs outside the living body using large form factor 3D bioprinters. To deliver 3D‐printed living constructs into the body, a large‐open field surgery is mostly required. The implantation process of 3D‐printed live constructs is at present severely limited by many factors. For example, the implantation of a cardiac patch for repairing damaged myocardium requires large open‐chest surgery, which increases infection risk. Treatment of gastric wall injury such as bleeding by spraying styptic colloid using an endoscope is not able to reconstruct the 3D structure of the wound. Although other injuries requiring resection are nonuniform in size and shape, the fabrication of in vitro 3D construct to match the 3D surface of the defect structure (e.g., stomach, colon) is challenging, making in situ printing an excellent approach to address this issue. This is due to the fact that precise control of the final shape, size, and gelation time of 3D printed live constructs is difficult and this in turn leads to an irregular shape, nonuniform macrostructures, and inconsistencies in functionalities, limiting their therapeutic potential. Minimally invasive delivery of biomaterials using current 3D bioprinters is still a distant possibility. Although manual handheld printing tools could perform in situ bioprinting, they are not able to perform complex shapes, and have low accuracy, while their physical constraints are limited to damaged areas of external and near the skin surfaces or sites exposed by large open surgery such as bone or cartilage. In this work, we have introduced a new concept of a flexible 3D bioprinter where biomaterials can be directly delivered into the target tissue or organs with a minimally invasive approach. We have introduced the design and conducted experimental validation of a miniaturized and multifunctional 3D bioprinter that can 3D print multilayered biomaterials of different sizes and shape through confined and hard‐to‐reach areas, thanks to its flexible body. The new F3DB was ex vivo validated with various printing patterns and biomaterials such as food‐grade chocolate, composite gel, and biomaterials on both flat, naturally curved surfaces and artificial colon instead of performing in vivo tests on living animals. In addition, the new device could be used as a multifunctional endoscopic tool that could perform necessary surgical steps for ESD procedures such as water jets, marking lesions, and tissue dissection. Using a master‐slave architecture, kinematic inversion model, and advanced motion controller, the F3DB could perform precise printing and tissue dissection at a remote distance under the presence of nonlinear hysteresis and disturbances. Compared to the large form factor of desktop 3D bioprinters which are normally large, rigid, and enclosed by sturdy frames (as shown in Table S1, Supporting Information), our technology has distinct advantages as it was designed with a miniature and snake‐like flexible body that is well adapted to confined space while offering a minimally invasive manner. Currently, there are no commercially available devices that can perform in situ 3D bioprinting. Although recent proof‐of‐concept in situ bioprinters has been introduced, they are either limited to rigid components, bulky driving sources, or poor reachability and bendability to work against complex and confined spaces.^[^ ^5^, ^13^ ^]^ In contrast, our printing tip could offer at least a 100° omnidirectional bending workspace for coarse navigation toward the region of interest. Using an advanced control algorithm, our 3D printing head also enables fine movements to enable precise printing profiles and tissue dissection. To provide a larger printing area, our printing tip can be expanded for multisite printings which effectively cover the target surface of an organ or tissue via an appropriate steering and mapping process. Compared to endoscopic robotic suturing, the 3D bioprinting approach offers a safer and simpler solution than the conventional surgical suture for promoting wound healing.^[^ ^27^ ^]^ Our F3DB with an in situ bioprinting method can also revolutionize the closure of mucosal defects after performing ESD compared to a variety of clips, fastening elements,^[^ ^2a^ ^]^ or the endoscopic suturing device.^[^ ^28^ ^]^ Compared to the existing endoscopic surgical tools, the developed F3DB was designed as an all‐in‐one endoscopic tool that avoids the use of changeable tools which are normally associated with longer procedural time and infection risks.

Although the idea of using fluid‐driven actuators arranged in parallel for multidirectional bending and elongation has been introduced in the literature,^[^ ^29^ ^]^ there are limited approaches to exploiting fabric actuators for surgical robotics as we presented here. Our FBA and SMAM designs are scalable for both length and size as well as stiffness using a different type of elastic inner tube and fabric or wrinkling percentage. The movable mechanism was designed as a separate module with customized hollow tubes and acrylic elements, which can be mass‐produced using additive manufacturing such as laser micromachining or 3D printing to meet the required sizes and scales. In addition, the printing head with a moving nozzle can be also customized for different medical uses such as electrospinning^[^ ^30^ ^]^ or a fiber‐delivered laser.^[^ ^31^ ^]^ The outer sheath mechanisms are useful in guiding the SMAMs and preventing them from buckling, it also contributes to constraining the movements of the bending robotic arm and enhances the arm stiffness, which limited the bending angle of the arm to less than 100^°^ all in directions. Therefore, future work should consider rearranging and redesigning the outer sheath mechanisms for a better bending angle (at least 180^°^). Although many endoscopic robotic systems have been introduced,^[^ ^32^ ^]^ they have mostly been limited in application due to high cost, bulky footprint, complex maintenance process, and lack of multifunctional tools. In addition, most surgical instruments for ESD require strict sterilization in order to avoid contamination risk, introducing additional complexity and resource‐demanding compliance as well as a higher risk of device damage. Our developed F3DB system, in contrast, potentially provides a low‐cost disposable solution as most of the robotic components are made from off‐the‐shelf materials.

We have also introduced new nonlinear hysteresis model‐based feedforward and machine learning‐based controllers in order to enhance the position‐tracking performance of the printing system. The nonlinear hysteresis model has several advantages such as fewer model parameters compared to the asymmetric hysteresis Bouc–Wen model^[^ ^22^, ^33^ ^]^ or a simpler form than the latest hysteresis model developed in our previous work.^[^ ^15^ ^]^ As a result, it requires less computational time and less complicated control processes. It is worth noting that the hysteresis profile and the offline‐learning controller vary under the change of external loads or disturbances. Although the 3D printing head has operated under a constant load condition, disturbances during the operation might enlarge tracking errors. To achieve higher accuracy in line with the repeatability of the device, real‐time sensing feedback is highly desired. A simple approach is to embed a soft strain gauge^[^ ^34^ ^]^ or optical fiber sensors^[^ ^35^ ^]^ into the SMAM to estimate the artificial muscle position and force.^[^ ^36^ ^]^ A nonlinear adaptive control algorithm can then be presented to deal with the disturbances and uncertainties.^[^ ^37^ ^]^ Measuring the movable mechanism position directly would result in a better position estimation, but integrating such a sensing method is not straightforward due to spatial and medical restrictions.^[^ ^38^ ^]^ Alternatively, visual feedback available from the flexible camera with data‐driven algorithms could be used for high‐quality real‐time estimates. In practice, the combination of sensory feedback for SMAM and visual feedback for the movable mechanism as well as a nonlinear adaptive control algorithm is likely the best approach.

It is noted that the adherence and reliability of the 3D printed construct to interact with internal tissue and organs highly depend on the biomaterials used, which are not the main focus of this work. However, in this study, we used di‐tyrosine crosslinking chemistry to demonstrate the feasibility of F3DB devices for 3D bioprinting toward clinical applications. This di‐tyrosine chemistry is based on the tyrosine residues that are found in the extracellular matrix and are identified in several materials including silk, gelatin, elastin, and keratin. This di‐tyrosine crosslinking method has been exploited its potential in in situ applications for clinical applications by several studies. In a recent study, photocrosslinked silk using the same di‐tyrosine crosslinking chemistry was used for laparoscopic surgery in a laceration rabbit model of liver and stomach serosa using a homemade endoscopic device.^[^ ^39^ ^]^ The printed constructs adhered to the native tissues as an adhesive wound dressing. Therefore, we hypothesize that this bioink, GelDAT, could also have adherence properties due to the crosslink to some degree between GelDAT and native tissues. Thus, in vivo studies will be our next focus in future work.

Despite advances, there are several areas within the F3DB system that can be improved in future works. First, the kinematic inversion model was proposed using the geometric relation of the structure, which has not accounted for the velocity of the movable mechanism. Therefore, the incorporation of such parameters into the kinematic model is highly desired. Second, the developed bending arm with an integrated camera has not been fully demonstrated. Therefore, the combination of the flexible miniature robot as presented in our previous work^[^ ^15^ ^]^ with a CMOS camera would be a great addition. Third, a real‐time scanning system has not been developed to reconstruct the 3D tomography of the moving tissue. Although it is out of the scope of this work, which has been focused mostly on the design, fabrication, and control of the F3DB, the integration of a micro‐optical fiber system and an external laser source into the printing head will beneficially provide real‐time detection of moving tissue, which will be then utilized to develop a closed‐loop feedback control. Therefore, in vivo test of the new F3DB with living animals should be conducted in the future to demonstrate its practical use.

In a nutshell, we have introduced a novel F3DB that can potentially deliver biomaterials in situ for faster restoration and healing of damaged tissues/organs. This new system, based on novel soft robotic technologies and advanced control algorithms, will be a disruptive change from the current large form factor of desktop 3D bioprinters and manual handheld bioprinters with minimally invasive procedures. It will also open new approaches in the development of the next generation of flexible surgical robots and medical devices using soft robotic technologies.

## 4 Experimental Section

### Mechanical Response Evaluation—Workspace Evaluation

The motion capability (the most important feature of the robotic arm) was first evaluated. The experimental setup using a high‐resolution camera was applied to capture various motions of the slave manipulator, while FBAs were inflated independently by the actuation blocks over a safe pressure range. Figure S7 and Movie S4 (Supporting Information) show that the bending angle of the arm toward left, right, forward and backward directions could exceed 100^°^. Next, the working area of the movable mechanism was assessed to reach a targeted position in the desired direction as shown in Figure S8, Supporting Information. It is noted that the movable mechanism could reach a circle of 13 mm within the working area of 17 mm in diameter due to its 4 mm diameter. This demonstrates that the movable mechanism could accomplish 76.5% of the theoretical working area. Furthermore, the movable mechanism was always upright (up to 6 mm in extension) when it was driven by four SMAMs toward any direction due to the design of flexible transmission tubes and curved metal tubes.

### Mechanical Response Evaluation—Force Capabilities

Experiments were also carried out to test the bending force at the nozzle of the 3‐DOF soft slave manipulator, the shear force, and normal force at the movable mechanism within the 3D printing head using a miniature FUTEK load cell (1 lb, FUTEK, USA) connected to a fixed structure. The robotic arm was mounted close to the load cell and bent when elongating the single FBA or dual FBAs and then the generated force was recorded. Figure S9a (Supporting Information) shows the generated force in six directions after five trials under the same conditions. The bending force was different in three principal directions due to inherently inconsistent fabrication, imperfect assembly, and friction between FBAs, which is not a big issue because the robotic arm is regulated by a surgeon via the master console. The maximum force generated by the slave manipulator is 1.31 N and the maximum standard deviation of bending force is 0.09 N, so the robotic arm manipulator generated fairly consistent forces. To evaluate the capability of the movable mechanism in generating force for performing any surgical tasks, the shear forces in eight directions and the normal force were measured in five trials and their results are shown in Figure S9b (Supporting Information). The data showed that the shear forces along diagonals were smaller than the principal directions of the 3‐axis printing head because two adjacent SMAMs produced a higher force compared to a single SMAM. The shear forces in the four main directions were slightly dissimilar because of inherent inconsistencies in the fabrication process by hand and also due to measurement errors. This unexpected factor might be dealt with by using a hysteresis model‐based feedforward controller for force control. Furthermore, the repetitive force accuracy of the movable mechanism was very high during the repeated (*n* = 5) of elongation and contraction of the SMAMs. The maximum force generated by the 3D printing head was 0.91 N and the maximum standard deviation of the generated force was 0.08 N.

### Mechanical Response Evaluation—Frequency Response

The dynamical response of the movable mechanism under hydraulic actuation was tested. A SMAM was extended or contracted through a hydraulic syringe actuated by an actuation block. A 3‐m‐long hydraulic transmission tube was used to meet the transmission requirements between working locations and the motor housing. The positional output of the printer nozzle under periodic sinusoidal input from 0.1 to 5.2 Hz was captured by a laser sensor (Keyence, model IL‐100, Keyence Corp., USA) as shown in Figure S10a (Supporting Information). The positional output for five testing cycles was collected and then calculated its mean and standard deviation at each frequency. The experimental result (Figure S10b, Supporting Information) showed a steady decrease in signal magnitude. At 5.2 Hz, the movable mechanism has lost its amplitude by −0.85 dB. This means that the changing frequency of the positional command should be more than 5.2 Hz without filtering due to the −3 dB cut‐off frequency. The result also revealed that the movable mechanism responded well to high‐frequency input signals, potentially benefitting printing applications. the typical −3 dB cut‐off frequency because of mechanical system limitations was unable to reveal. Furthermore, the transmission latency or time delay from the computer signal to distal output was measured as 103 +/−20 ms, which was considered in the control algorithm.

### Mechanical Response Evaluation—Durability

The reliability of the movable mechanism should be maintained in a prescribed amount of time despite the intention of single use. As shown in Figure S10c (Supporting Information), the printer nozzle was moved forward and backward repeatedly by actuating a SMAM for 1000 cycles over 1.4 h. The printing head remained functional. While the absolute printer‐prescribed motion decreased by only 13.8%, the pressure decreased by 11.2% over the testing period.

### Biomaterials with Living Cells—Bioink Preparation

GelDAT (Rousselot) was dissolved in 0.9% NaCl to get a 10% GelDAT solution. The solution was sterilized using a 0.2 µm filter membrane before mixing with the cell suspension. L929 cells (85 011 425, Sigma‐Aldrich, New South Wales, Australia) were then cultured in Dulbecco's Modified Eagle's Medium‐high glucose supplemented with 10% Fetal Bovine Serum and 1% penicillin‐streptomycin (PS). At 70–90% confluency, cells were rinsed twice with sterile PBS and incubated in TrypLE Express at 37 °C, 5% CO_2_ for 5 min to detach cells from the surface. After 5 min, the cell suspension was collected and centrifuged at 200 rpm for 3 min to obtain cell pellets (Centrifuge 5810 R, Eppendorf, Australia). The cell pellets were then resuspended in supplemented DMEM‐high medium and counted using a cell viability analyzer (Vi‐cellXR, Beckman Coulter, Australia). The cell suspension was further mixed 1:3 volume ratio with 10% GelDAT solution to obtain the precursor of bioink with 1.25 × 10^6^ cells mL^−1^ in final cell density. Next, the bioink precursor was added to photoinitiators, Ruthenium (Ru) and Sodium Persulfate (SPS), at a final concentration of 0.5 and 5 mm, respectively. The final bioink was covered with aluminum foil to prevent it from being precrosslinked before printing. More information on bioink can be found in Note S6 (Supporting Information).

### Biomaterials with Living Cells—3D Printing Cell‐Embedded Construct

The bioink was loaded into the F3DB and printed through a needle of 25G at a flow rate of 100 µL min^−1^. The printing was performed in a standard 24‐well tissue culture plate. The printed patterns were in situ crosslinked immediately after extruding from the nozzle using a 30 W LED array portable light/work lamp (Jobmate, New Zealand) as described elsewhere.^[^ ^40^ ^]^ At the end of the printing process, all the printed rectangles were further crosslinked under the 30 W LED array portable lamp (Jobmate, New Zealand) for 3 min before being submerged in supplemented DMEM‐high medium. All the printed constructs were cultured at 37 °C with 5% CO_2_. The printed cells were analyzed for cell viability and proliferation over 7 days using the Alamar blue assay and live/dead fluorescent staining as per the manufacturer's instructions.

## Conflict of Interest

The authors declare no conflict of interest.

## Supporting information

Supporting Information

Supplemental Video 1

Supplemental Video 2

Supplemental Movie 3

Supplemental Movie 4

Supplemental Movie 5

## Data Availability

The data that support the findings of this study are available from the corresponding author upon reasonable request.
