# Supplementary material for: Advanced Soft Robotic System for In Situ 3D Bioprinting and Endoscopic Surgery
Source: Adv Sci (Weinh). 2023 Feb 19;10(12):2205656. doi: 10.1002/advs.202205656 (PMC10131836; doi:10.1002/advs.202205656)
Supplement: Supplementary file 1 — Supporting Information [file ADVS-10-2205656-s004.pdf]

ADVANCED SCIENCE
Open Access

Supporting Information

for *Adv. Sci.*, DOI 10.1002/advs.202205656

Advanced Soft Robotic System for In Situ 3D Bioprinting and Endoscopic Surgery

*Mai Thanh Thai, Phuoc Thien Phan, Hien Anh Tran, Chi Cong Nguyen, Trung Thien Hoang, James Davies, Jelena Rnjak-Kovacina, Hoang-Phuong Phan, Nigel Hamilton Lovell and Thanh Nho Do**

Supporting Information

Advanced Soft Robotic System for In Situ 3D Bioprinting and Endoscopic Surgery

*Mai Thanh Thai, Phuoc Thien Phan, Hien Anh Tran, Chi Cong Nguyen, Trung Thien Hoang, James Davies, Jelena Rnjak-Kovacina, Hoang-Phuong Phan, Nigel Hamilton Lovell and Thanh Nho Do**

Mai Thanh Thai, Phuoc Thien Phan, Hien Anh Tran, Chi Cong Nguyen, Trung Thien Hoang, James Davies, Jelena Rnjak-Kovacina, Nigel Hamilton Lovell and Thanh Nho Do
Graduate School of Biomedical Engineering, Faculty of Engineering, UNSW Sydney, Kensington Campus, NSW 2052, Australia
Tyree Institute of Health Engineering, UNSW Sydney, NSW 2052, Australia
E-mail: tn.do@unsw.edu.au

Hoang-Phuong Phan
School of Mechanical and Manufacturing Engineering, Faculty of Engineering, UNSW Sydney, Kensington Campus, NSW, 2052 Australia
Tyree Institute of Health Engineering, UNSW Sydney, NSW 2052, Australia

Supplementary Table S1. Summary of various mechanisms for in vivo bioprinting

**Type**	**Printing depth**	**Incision size**	**Printing area**	**DOF**	**Accessible to natural orifices**	**Disposability Low-cost**	**Number of functions**
Conventional method [1]	Surface (Epidermal)	Traditional surgery	-	One	No	No	Single
Near-infrared polymerization [2]	<10 mm (Subcutaneous Tissue)	Non-invasive	<0.2 cm^2	Three	No	No	Single
Micro bioprinting platform [3]	>100 mm (Inside body)	~28 mm (minimally invasive)	~ 9 cm^2	Three	No	No	Single
FSCR printing system [4]	0 - 150 mm (Inside body)	2~4 mm (minimally invasive)	0~27 cm^2	Three	No	No	Single
**F3DB**	**>500 mm (Inside body)**	**~20 mm through natural orifice**	**0~30 cm^2**	**Six**	**Yes**	**Yes**	**Multiple**

Supplementary Table S2. Motion range of the components in the F3DB.

No.	Motion	Soft robotic arm	Slave manipulator	3D printing head
1	Bending	$-120^o \rightarrow 120^o$	$-100^o \rightarrow 100^o$	None
2	Extension	0 → 20 mm	0 →18 mm	6 mm
3	Working boundary (in diameter)	65 mm	60 mm	13 mm

Supplementary Table S3. Geometric parameters of the 3D printing head for the inverse kinematic model

l_0	h	$\emptyset_D$	$\emptyset_d$
30 mm	8.5 mm	19 mm	4 mm

Supplementary Table S4. Geometric parameters of the robotic arm for the kinematic model

l_0 (Orignal length of the robotic arm)	h	d
20 mm	25 mm	4 mm

Supplementary Note 1. The motor housing

The motor housing is the brain of the robotic system consisting of a controller board (QPIDe Data Acquisition Device, Quanser, Canada) and connecting to a computer with software, nine actuation blocks containing nine DC motors (model 3272G024CR, Faulhaber, Germany) with nine a translational ball screw mechanism (MISUMI, Japan) and other electronic components (e.g., IO circuits, power suppliers, and drivers). While five actuation blocks are used for controlling the printing head, three actuation blocks actuate the 3DOFs of the soft robotic arm. The last actuation block regulates the printing material flow. A detailed actuation block is shown in [5]. There is a high-resolution encoder in each DC motor to make sure highly accurate position control which is advantageous for a feedforward controller and a learning-based controller mentioned in this article. Because the robotic arm located at the tip of the system is actuated by fluid-driven soft actuators and its driving source is located outside the human body, each actuation block actuates a syringe plunger in a BD Luer-Lok™ 1 mL syringe to apply fluidic pressure to the soft actuators through a hydraulic transmission tube. This energy transmission method has a constant energy efficiency regardless of the transmission paths in length and configuration which is suited to endoscopic applications [5] as compared to tendon-driven mechanisms where there is a high variation in energy transmission through a long distance. The software written in MATLAB Simulink (Mathworks, Inc., USA) processes signal communication between the master console and the motor housing while

executing any instructions from the surgeon. The system framework is built with two-layered architecture. The low-level layer manages hardware control and position control for each DC motor while the high-level layer performs the signal processing to compensate for hysteresis of each soft actuator and position control for the tip of robotic arms.

Supplementary Note 2. Kinematic model of the soft robotic arm

The forward and inverse kinematic model for extensible multi-backbone robotic robots has been formulated and presented in [6]. Inspired and adapted by these works, this paper describes a closed-form kinematic model using the length of each soft fabric bellow actuator (FBA) as the actuator space and the robotic arm tip as the task space. The length of each actuation element will then be controlled by the feedforward controller presented in the paper. This means we can control the position and direction of the printing nozzle based on the length of FBAs. There are two assumptions for this model: all center lines of actuation elements are parallel at any working state due to mechanical constraints, and constant curvature is applied to all actuation elements (Figure S1a). The effect of external forces is not considered in the model due to 3D printing applications not involving any external forces. As illustrated in Figure S1b, a kinematic model was developed to capture the relationship between the tip/nozzle pose (position and orientation) and the length of each actuation element through two mappings. One is from the actuator space q (l_1, l_2, l_3) to the configuration space C (k, θ, φ), which defines constant-curvature arcs. The other is from the configuration space to the task space $[x, R]$ consisting of the position and orientation of the arm tip.

$$x = \left[T_x, T_y, T_z\right]^T, R = \ R_t, R \in SO(3) \tag{1}$$

$$C = [k, \theta, \varphi]^T \tag{2}$$

$$q = [l_1, l_2, l_3]^T \tag{3}$$

where x and R denote the position and orientation of the arm tip. $k, \theta,$ and φ represent the arc parameters of the robotic arm such as the curvature, the rotational angle about the +y-axis compared to the arm pose when all actuators are at rest, and the angle of the plane containing

the arc compared to +x-axis respectively. l_1, l_2 and l_3 denote the length of each actuation element at its centerline.

A closed-form relationship between actuator lengths and the resulting shape ($T_{specific}$) is expressed based on its geometry as:

$$l = \frac{l_1 + l_2 + l_3}{3} \tag{4}$$

$$k = \frac{2\sqrt{l_1^2 + l_2^2 + l_3^2 - l_1 l_2 - l_1 l_3 - l_2 l_3}}{d(l_1 + l_2 + l_3)} \tag{5}$$

$$\theta = kl \tag{6}$$

$$\varphi = tan^{-1}\left(\frac{\sqrt{3}(l_2 + l_3 - 2l_1)}{3(l_2 - l_3)}\right) \tag{7}$$

Where l represents the current length of the imaginary central backbone and d is the distance from the center of the imaginary central backbone to the center of the actuator, which is the same for all actuators. From Equation (7) it is possible to derive the bending direction angle based on the length of each actuation element which is controlled under an inverse model-based feedforward controller proposed in this paper.

The tip position vector x and rotation matrix R for the tip/nozzle of bending arms can be calculated by modifying a homogeneous transformation ($T_{independent}$) expressed in Equations (7) and (8) as:

$$T_{independent} = \begin{bmatrix} cos\varphi * cosks & -sin\varphi & cos\varphi * sinks & -cos\varphi * (\frac{cosks - 1}{k} - h * sinks) \\ sin\varphi * cosks & cos\varphi & sin\varphi * sinks & -sin\varphi * (\frac{cosks - 1}{k} - h * sinks \\ -sinks & 0 & cosks & h * cosks + \frac{sinks}{k} \\ 0 & 0 & 0 & 1 \end{bmatrix} \tag{8}$$

Where $s \in [0\ l]$ and h is the length of the arm tip or the combination between the printing head and the printing nozzle as shown in Supplementary Table S4. From Equations (4-8), we are able to regulate the position and direction of the arm tip by controlling each actuation element.

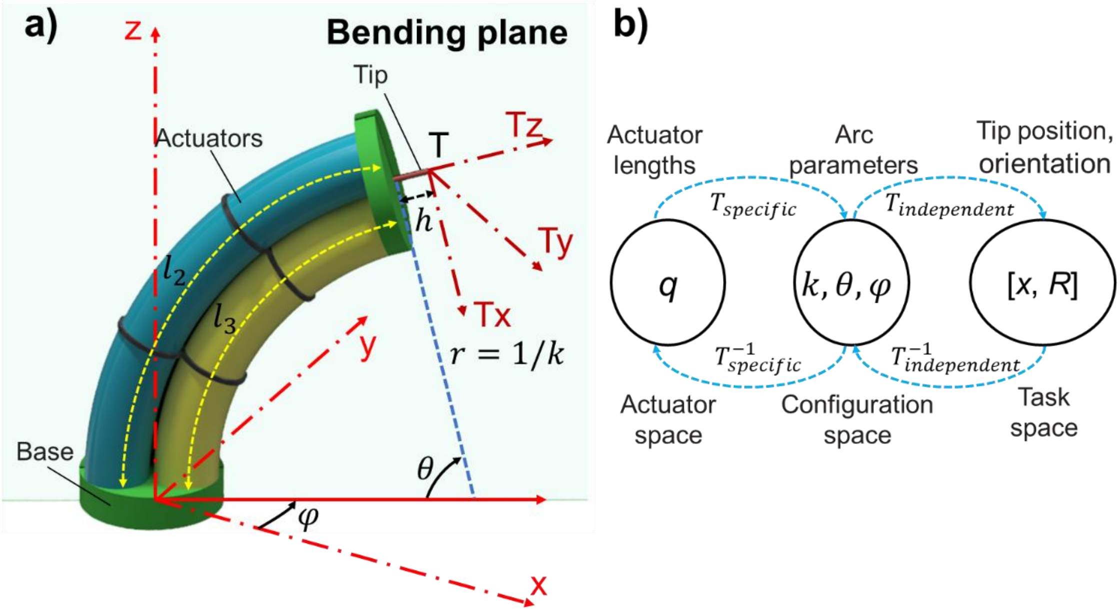

Figure S1. Kinematic nomenclature of the soft robotic arm. (a) The schematic of the soft robotic arm in three-dimensional space. (b) Mappings between three spaces of constant-curvature robots.

Supplementary Note 3. The fabrication process and assembly for the FBA and the 3-DOF soft robotic arm

Figure S2a highlights a fabrication process for each FBA based on a configuration of non-stretchable fabrics with two pairs of parallel stitches. Firstly, the non-stretchable fabric layers are aligned and stacked along their warp and weft. The fabric layers are then stitched (straight stitching type – 3 mm in each step) by a sewing machine (Brother FS155, Brother Industries, Japan) to form a conduit (3.5 Ø mm) between the fabric layers to allow tube insertion. In this design, we make one pair of stitches for each side of FBA to increase the whole structure's stiffness. To promote the radial expansion of the elastic tube when working, each piece of fabric should occupy an equal half of the tube diameter. Later, the residual fabric is cut and heated (at 200°C) along a cut edge 3 mm from the stitches to stop fabric edges from fraying. After sewing, the elastic tubes (Gecko Optical, Australia) are threaded through the conduits

via a needle with a thread attached to the end of the elastic tube. The fabric is then wrinkled (from 70 mm to 20 mm in length) along the length of the tubes to accommodate stretching. A uniform, controlled wrinkling can be achieved by hand after 10 minutes of training. Water is then pumped from a miniature syringe through a fluid transmission tube (Cole-Parmer, USA) into the elastic tube. A knot is made to seal one end of the elastic tube, while another end is sealed by polyester sewing thread and superglue (LOCTITE®, USA) to the fluid transmission tube. Finally, the elastic tube is bonded with the wrinkled fabric conduits at two ends to make the whole actuator deform together when it is in operation. Together, the wrinkling ratio and elastic properties of the fabric and elastic tube determine the maximum motion of the actuator.

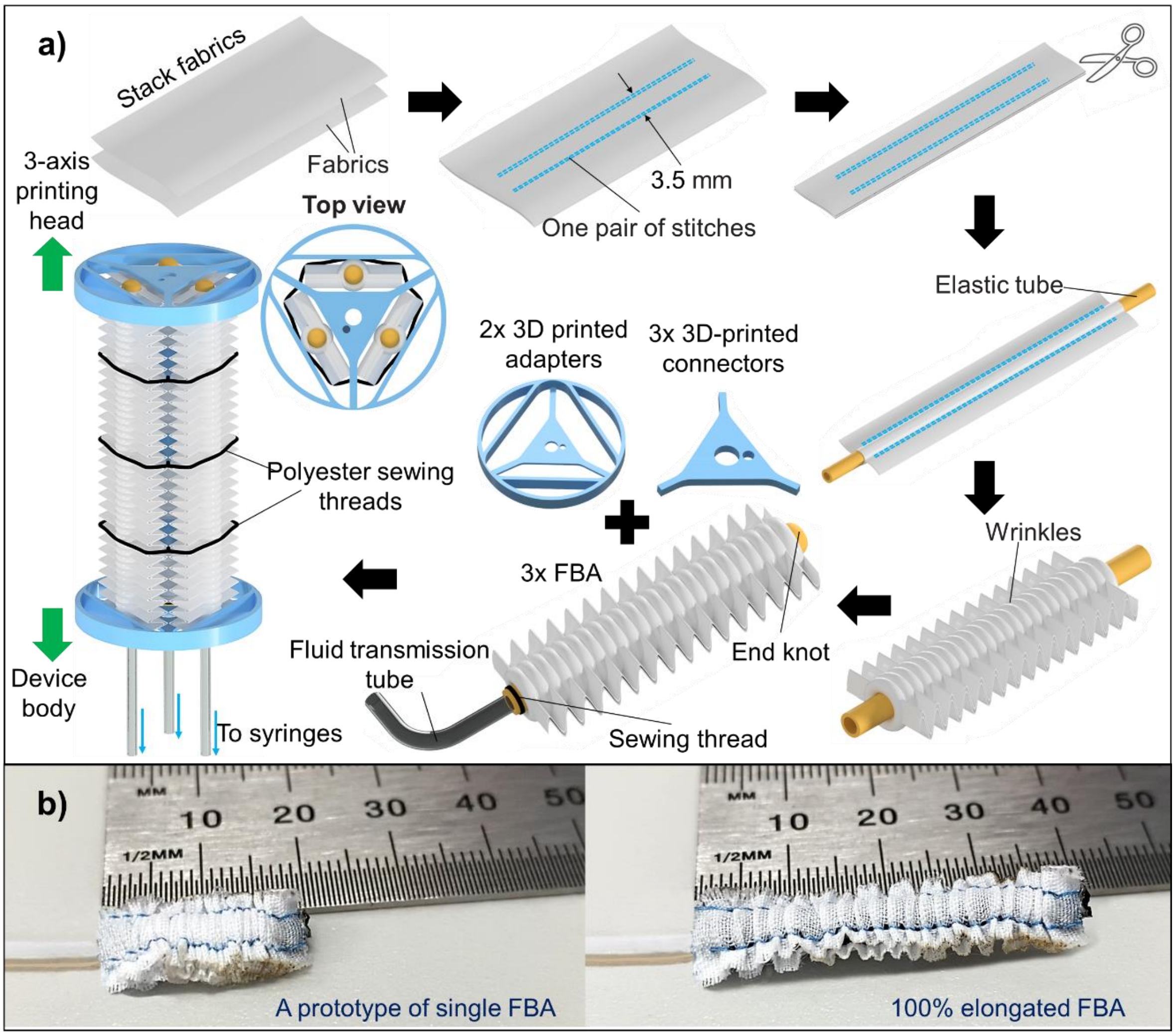

Figure S2. Schematic illustration of the fabrication process and assembly for the FBA and the 3-DOF soft robotic arm. (a) Fabrication process and assembly. (b) A prototype of the FBA at two pressurized states.

Supplementary Note 4. The fabrication process and assembly of some components in the 3D-printing head and the movable mechanism

At the beginning of the 3D-printing head fabrication process, the housing frame is 3D printed while the stainless-steel ring from a paper clip is formed as a circular frame by a jig (Figure S3b). Second, each SMAM is fabricated as shown in [5, 7], glued to its one end to heat shrink tubing and elongated to reach a level of 50% of its maximum strain so that a balancing force is maintained when they are connected. Third, SMAMs at their 50% maximum strain are glued to the middle acrylic element of the movable mechanism to form the 3-axis printing head. Fourth, little machine grease is added to reduce friction between SMAMs and the stainless-steel ring. Finally, a frame cap is installed to create enclosed space around SMAMs. The acrylic elements are created by using a laser cutting machine (Speedy 400, Trotec Laser GmbH, Austria) (Figure S3c). All elements were assembled using adhesive glue (LOCTITE®, USA) and polyester sewing thread.

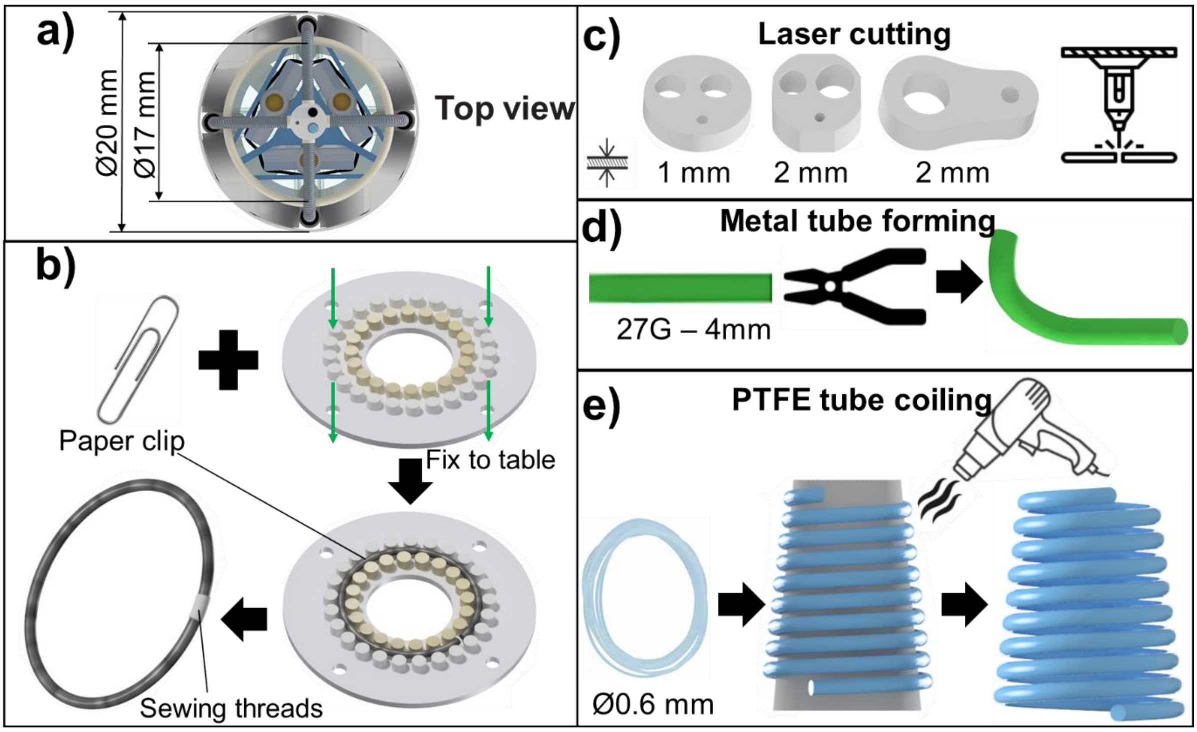

Figure S3. The fabrication process of the 3D-printing head. (a) Top view of the printing head. (b) A fabrication process of the stainless steel ring. (c-e) A fabrication process for three acrylic elements, curved metal tubes, and helical PTFE tubes, respectively.

Supplementary Note 5. Comparison between hysteresis models and experimental data

The hysteresis loops obtained from the conventional hysteresis Bouc–Wen model are symmetric in both the loading and unloading phases (Figure S4b). The symmetric Bouc–Wen model is not able to precisely follow the SMAM hysteresis profile with F = 0.4792 and the error is large near the middle of the hysteresis loop although there is a close fit at the transition points of the hysteresis loop. The generalized asymmetric Bouc–Wen model, in contrast, shows better results with a smaller value of F = 0.0946 (Figure S4c) because it is inherently able to follow asymmetric hysteresis loops. This model fits the experimental data not only near two transition points but also in the middle of the loading and unloading phases. The new proposed model given by Equation (6) and Equation (7), in contrast, offers the smallest F = 0.0636 compared to the other two well-known models (Figure S4a). In addition, it has seven model parameters, which is less than that of the generalized asymmetric Bouc-Wen model, resulting in less computational time. The hysteresis curve in the proposed model is asymmetric for the loading and unloading phases and it has good tracking properties in following the experimental data.

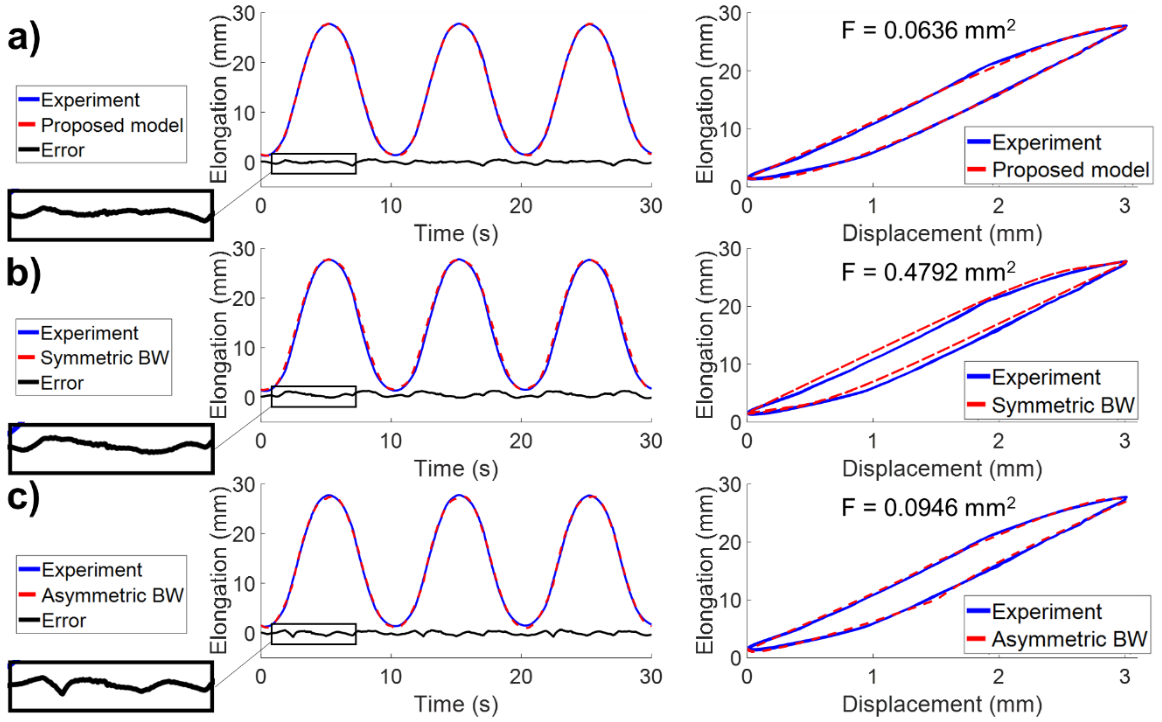

Figure S4. Comparison between hysteresis models and experimental data with 0.3 Hz sine wave input signal. (a) The proposed asymmetric hysteresis model. (b) The symmetric Bouc–Wen model. (c) The generalized asymmetric Bouc–Wen model.

Supplementary Note 6. Bioink preparation

Dulbecco's Modified Eagle's Medium-high glucose (DMEM-high), Fetal Bovine Serum (FBS), penicillin-streptomycin (PS), tris(bipyridine)ruthenium(ii) chloride (Ru), and sodium persulfate (SPS) were purchased from Sigma Aldrich Pty Ltd. (New South Wales, Australia). TrypLE™ Express, Alamar Blue, and LIVE/DEAD™ Cell Imaging Kit were purchased from Thermo Fisher Scientific Pty Ltd. (New South Wales, Australia). X-Pure GelDAT® 160D50RG powder (GelDAT) was adopted from Rousselot Biomedical Company.

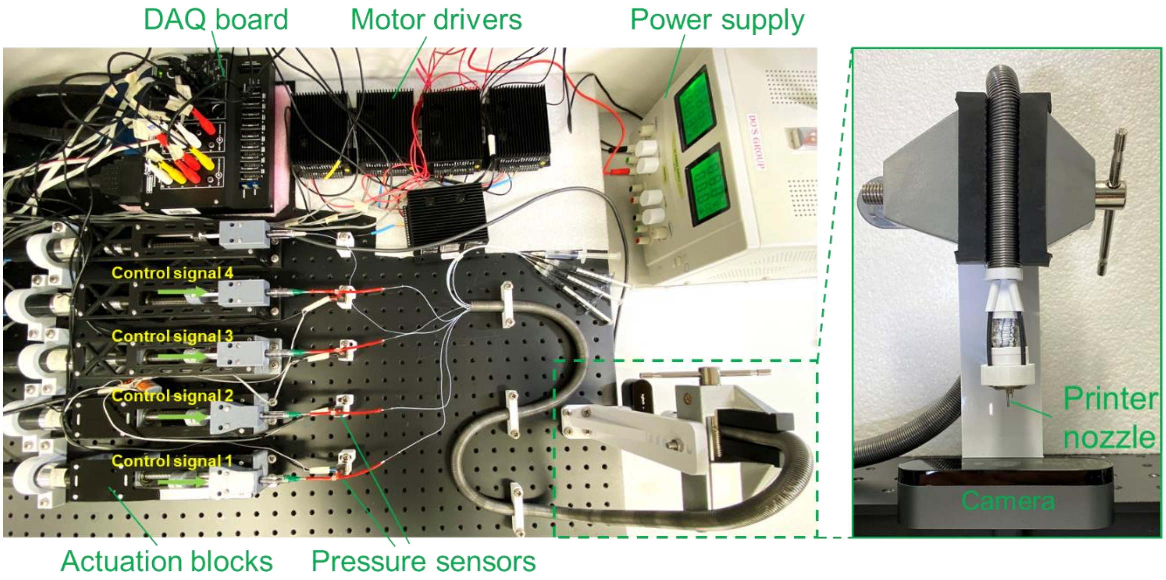

Figure S5. Experiment setup for the feedforward and learning-based controllers.

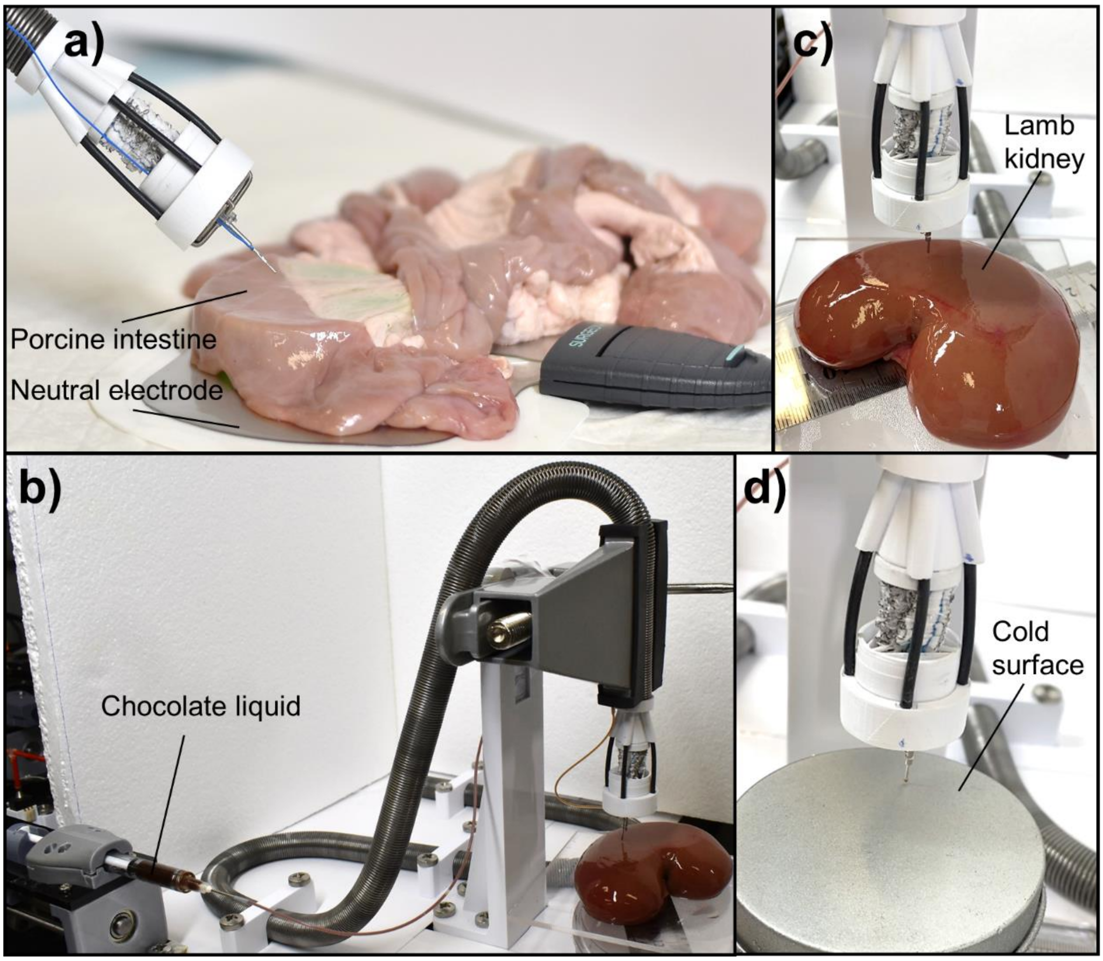

Figure S6. Ex-vivo experiment setup. (a) ESD procedure on the porcine intestine. (b) Experiment setup for 3D printing. (c) Ex vivo printing on lamb kidney. (d) Ex vivo printing on a cold surface.

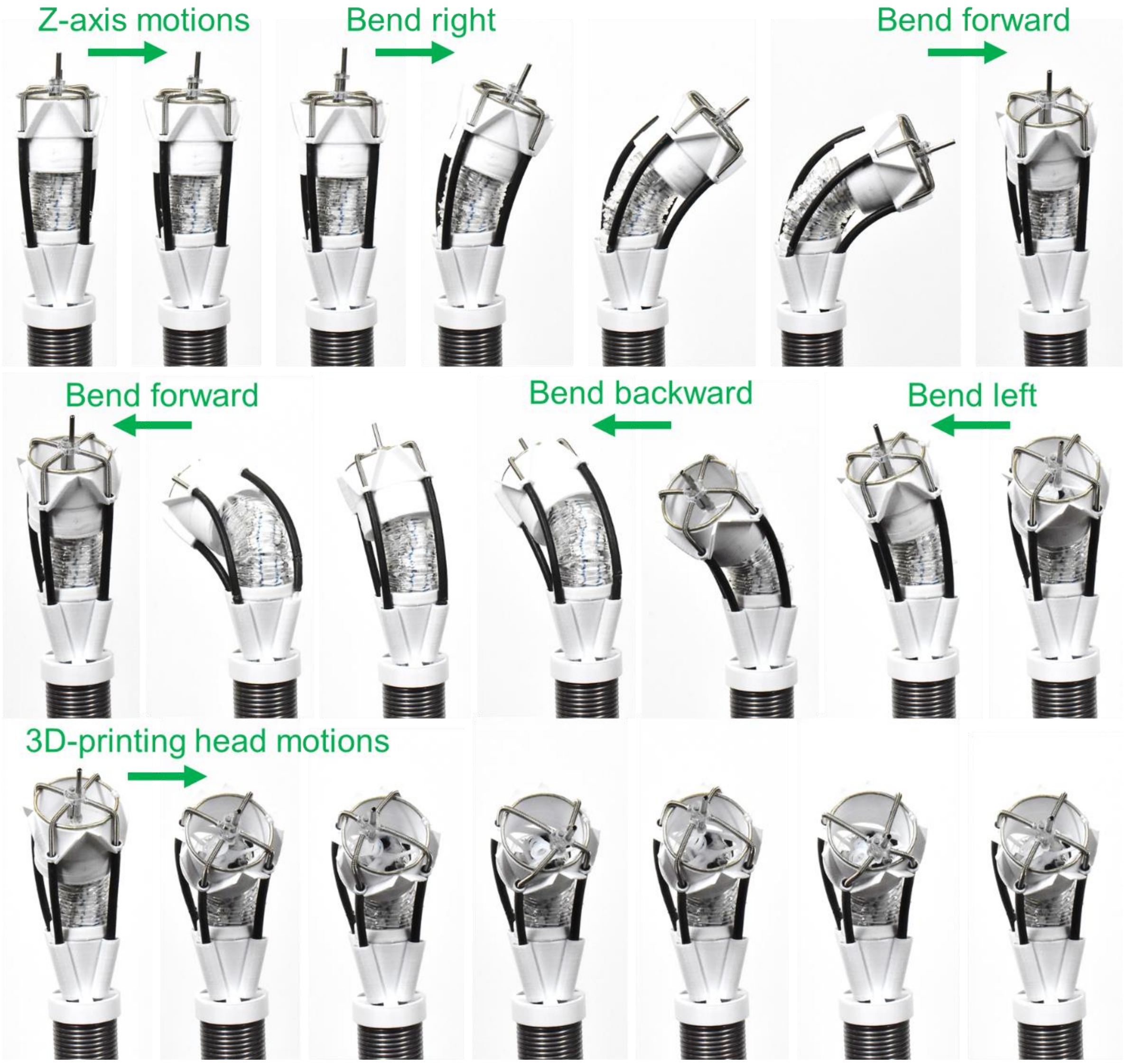

Figure S7. Cumulative image representing the bending motions of the 3D robotic arm with various positions of the 3D-printing head.

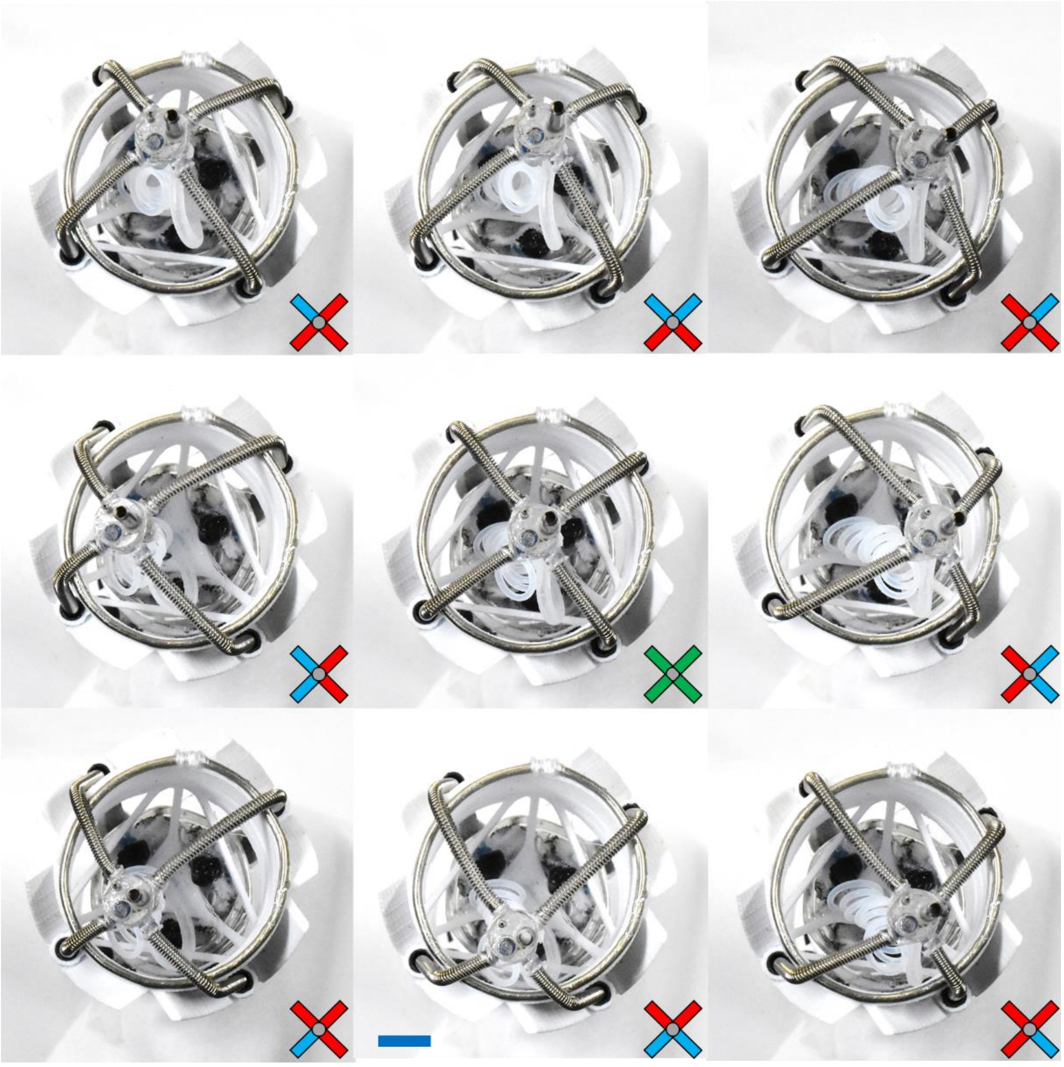

Figure S8. Various positions of the printing head with lengths of four SMAMs. In the bottom-right of each image, the pressurized muscles, the depressurized muscles, and the muscles in their initial state are shown in red, blue, and green respectively. Scale bar: 4 mm.

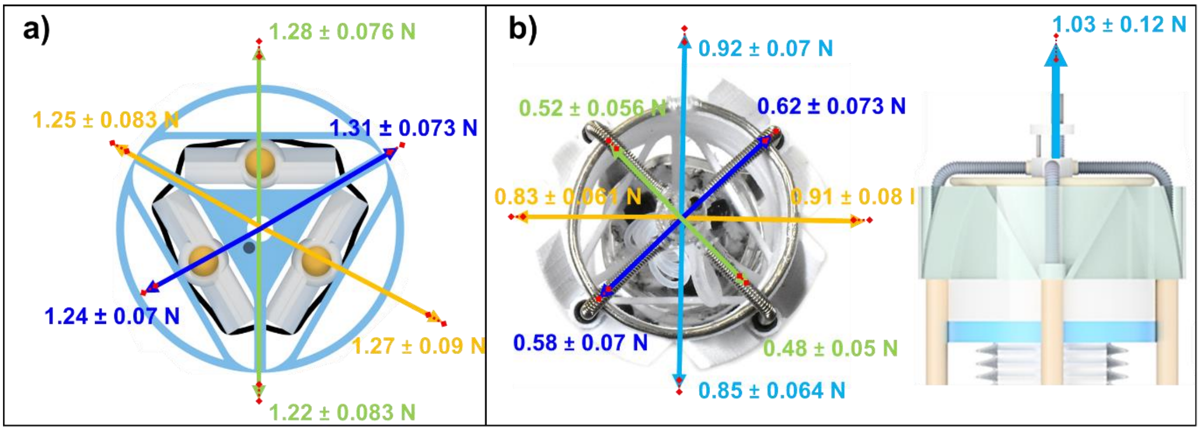

Figure S9. Force characterization of the 3D printing head at its nozzle. (a) Bending force at the nozzle of the 3-DOF soft robotic arm. (b) Shear force and normal force at the printing head.

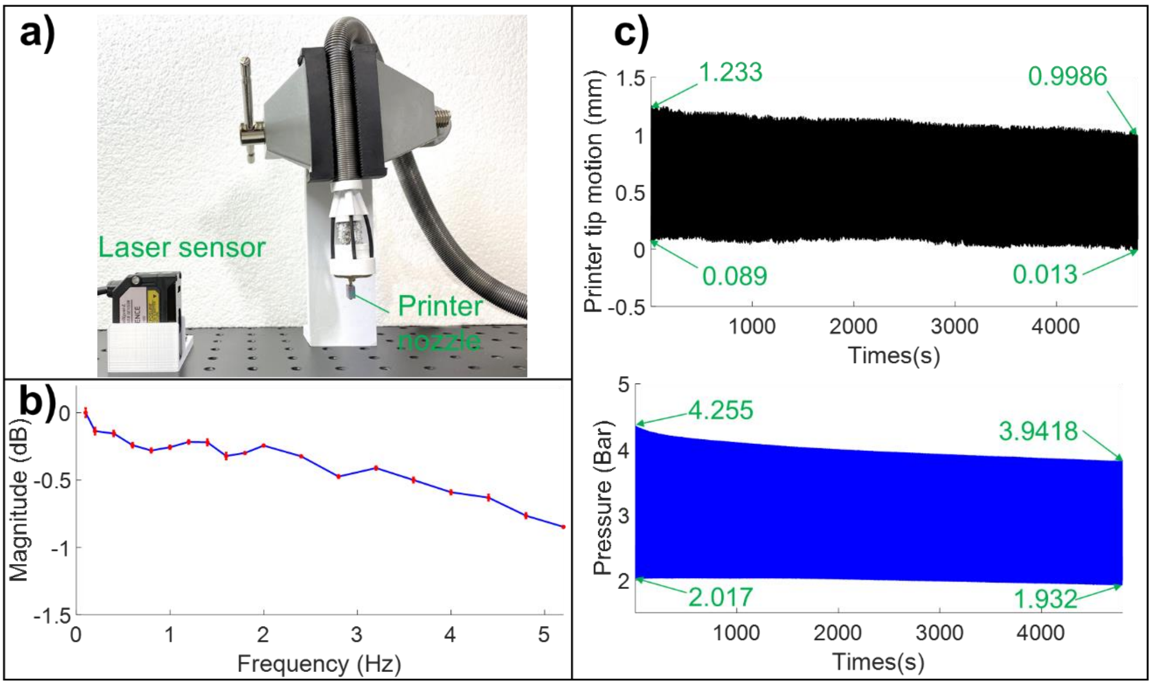

Figure S10. Mechanical performance test of the printing head. (a) Apparatus for mechanical testing. (b) Frequency responses of the SMAM up to 5.2 Hz in 5 cycles. (c) Printing head durability test performed by repeatedly actuating a SMAM 1000 cycles over 1.4 hours.

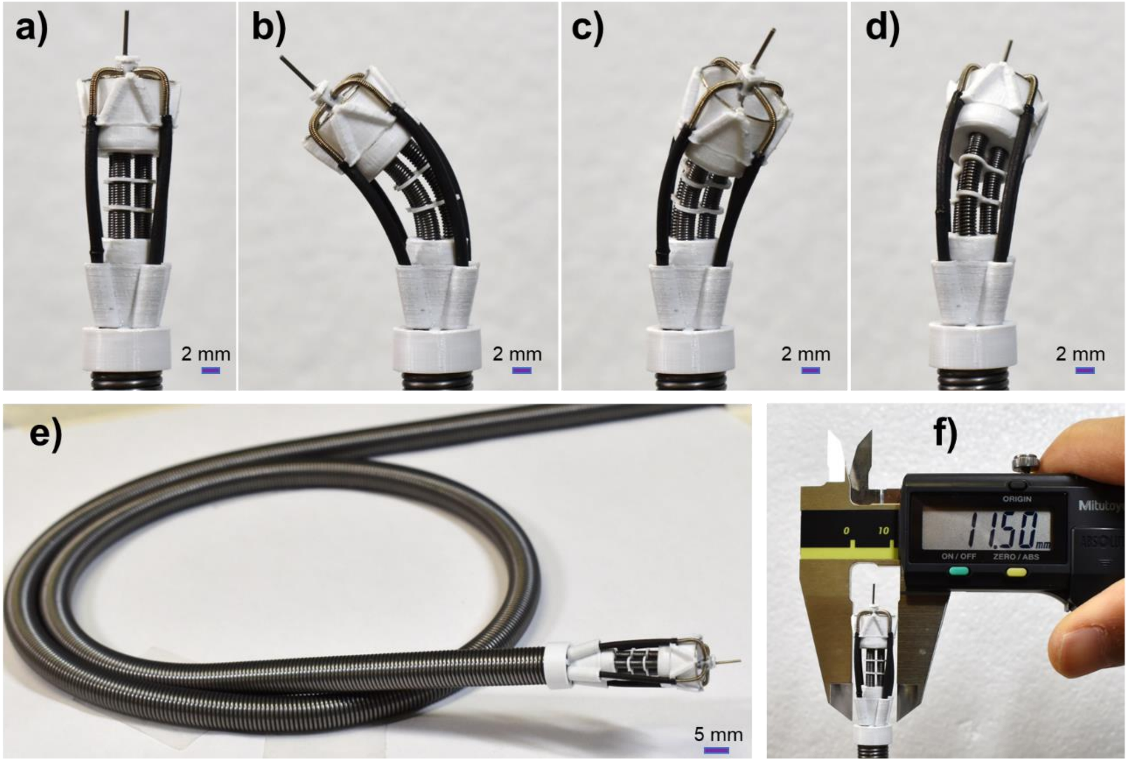

Figure S11. (a-d) Images representing the bending motions of the F3DB smaller version. (e) A prototype of the F3DB smaller version with its flexible body, 3D printing head, and flexible robotic arm. (f) The diameter of the prototype.

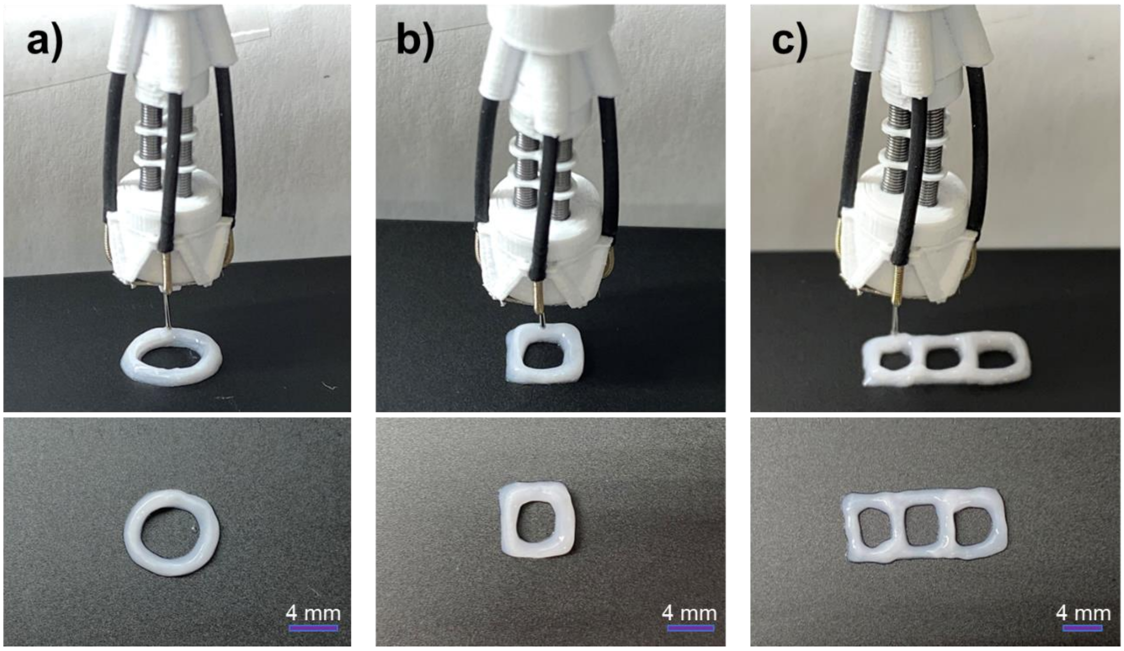

Figure S12. Printing performance of the F3DB smaller version with the gel composite. (a) Printing with five-layered circular shapes on a flat surface. (b) Printing with five-layered rectangular shapes on a flat surface. (c) Multisite printing on a flat surface when the F3DB is bending left.

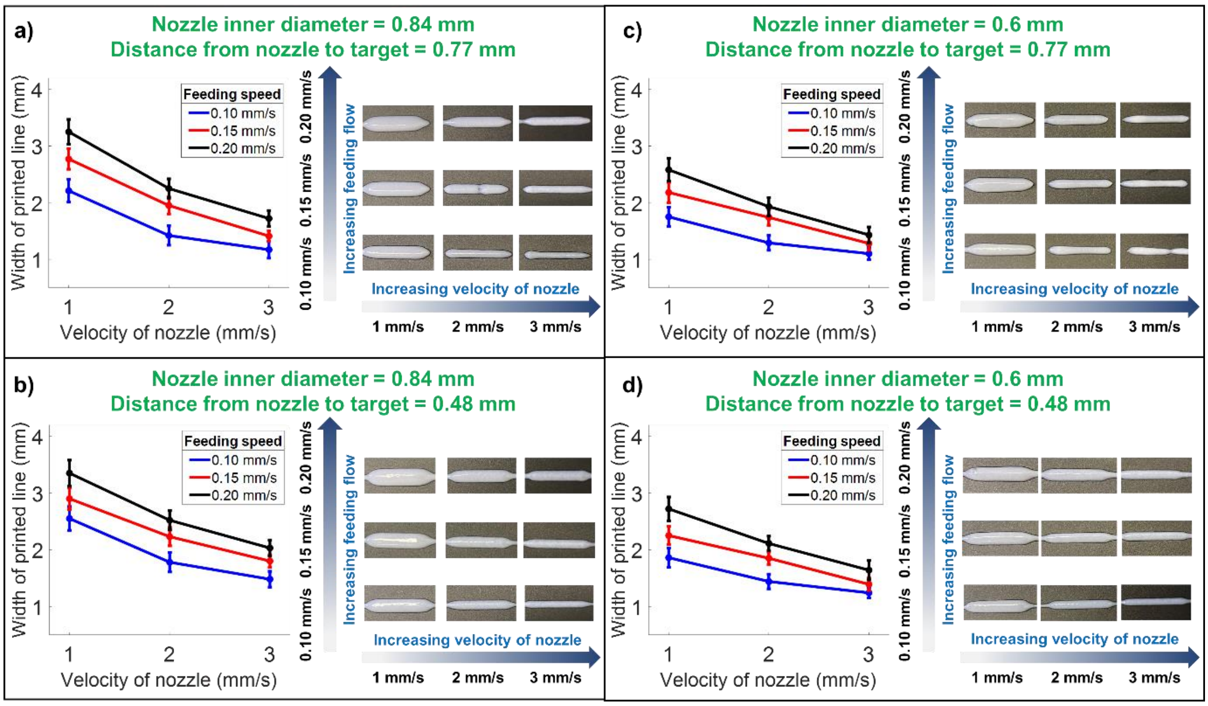

Figure S13. The width of the single-layer printed fiber. (a) The nozzle inner diameter was 0.84 mm and the distance from the nozzle to the target was 0.77 mm. (b) The nozzle inner diameter was 0.84 mm and the distance from the nozzle to the target was 0.48 mm. (c) The nozzle inner diameter was 0.6 mm and the distance from the nozzle to the target was 0.77 mm. (d) The nozzle inner diameter was 0.6 mm and the distance from the nozzle to the target was 0.48 mm. All the error bars represent standard deviation (n=5).

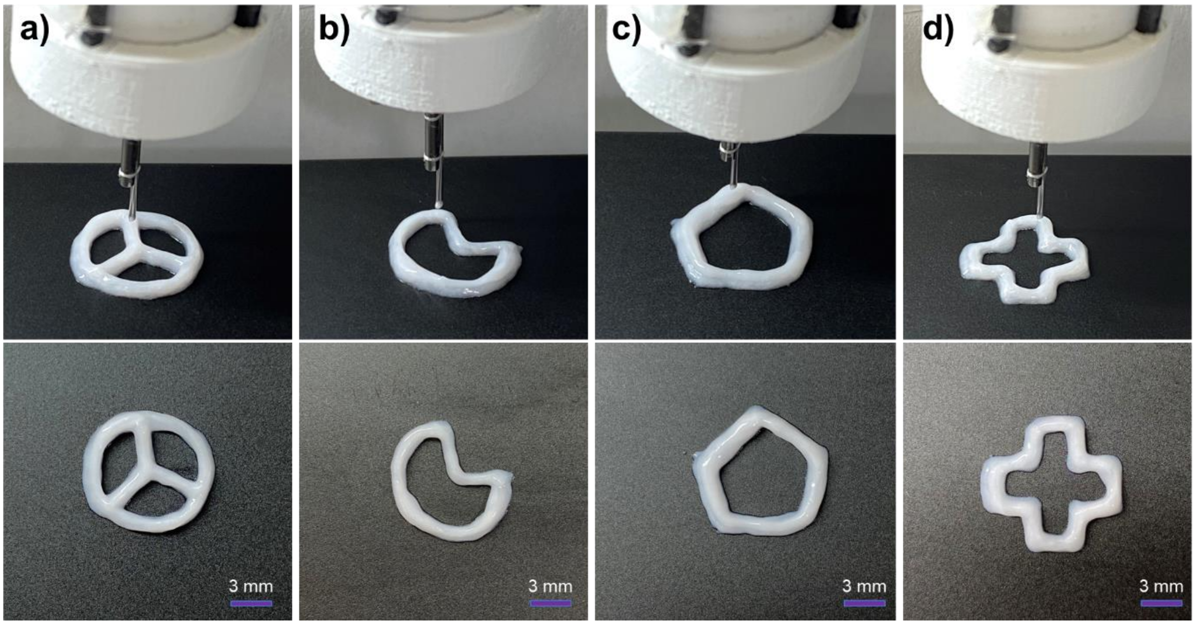

Figure S14. Printing performance of the F3DB with the gel composite. (a) Triradiate pattern. (b) Pie pattern. (c) Pentagon pattern. (d) Cross pattern.

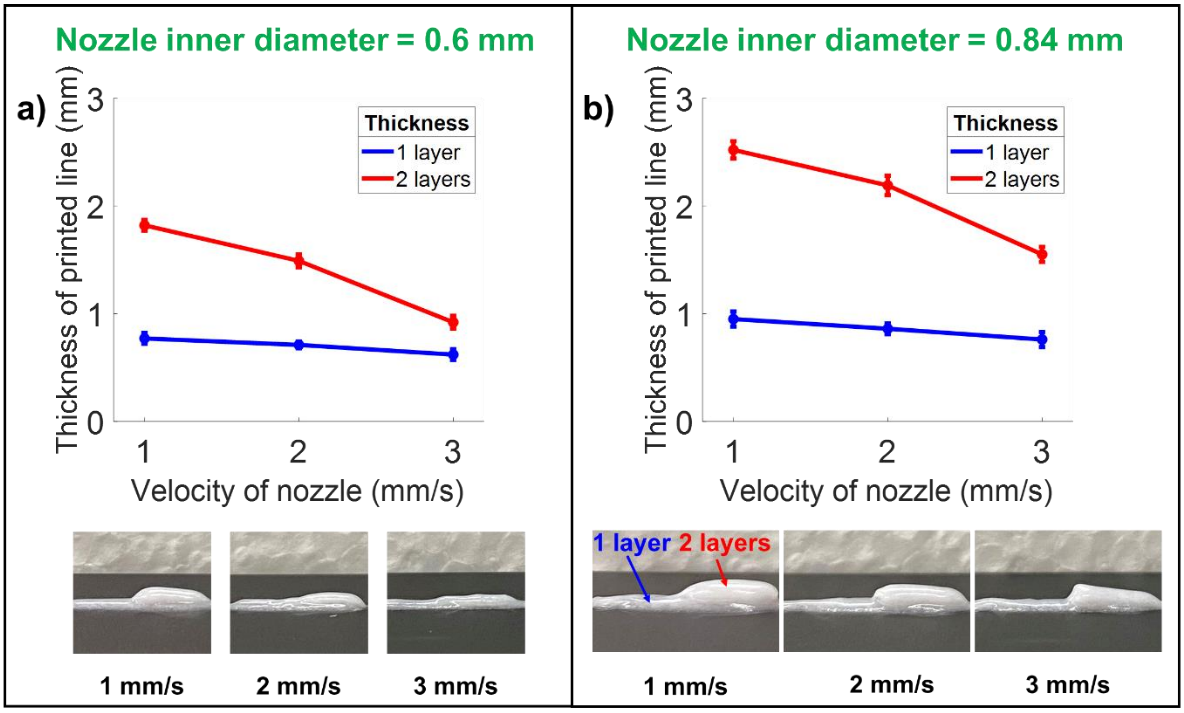

Figure S15. The thickness of the printed fibers. (a) The nozzle inner diameter was 0.6 mm. (b) The nozzle inner diameter was 0.84 mm. All the error bars represent standard deviation (n=5).

Other Supplementary Material for this manuscript includes the following: Supplementary Movies S1 to S5

References

[1] a) N. Hakimi, R. Cheng, L. Leng, M. Sotoudehfar, P. Q. Ba, N. Bakhtyar, S. Amini-Nik, M. G. Jeschke, A. Günther, *Lab on a Chip* **2018**, 18, 1440; b) Z. Zhu, S. Z. Guo, T. Hirdler, C. Eide, X. Fan, J. Tolar, M. C. McAlpine, *Advanced Materials* **2018**, 30, 1707495.

[2] A. Urciuolo, I. Poli, L. Brandolino, P. Raffa, V. Scattolini, C. Laterza, G. G. Giobbe, E. Zambaiti, G. Selmin, M. Magnussen, L. Brigo, P. De Coppi, S. Salmaso, M. Giomo, N. Elvassore, *Nat Biomed Eng* **2020**, 4, 901.

[3] W. Zhao, T. Xu, *Biofabrication* **2020**, 12, 045020.

[4] C. Zhou, Y. Yang, J. Wang, Q. Wu, Z. Gu, Y. Zhou, X. Liu, Y. Yang, H. Tang, Q. Ling, L. Wang, J. Zang, *Nat Commun* **2021**, 12, 5072.

[5] M. T. Thai, P. T. Phan, T. T. Hoang, H. Low, N. H. Lovell, T. N. Do, *IEEE Robotics and Automation Letters* **2021**, 6, 5089.

[6] a) S. Neppalli, M. A. Csencsits, B. A. Jones, I. D. Walker, *Advanced Robotics* **2009**, 23, 2077; b) R. J. Webster III, B. A. Jones, *The International Journal of Robotics Research* **2010**, 29, 1661.

[7] P. T. Phan, M. T. Thai, T. T. Hoang, N. H. Lovell, T. N. Do, *Ieee Access* **2020**, 8, 226637.
